# Grpel2 maintains cardiomyocyte survival in diabetic cardiomyopathy through DLST-mediated mitochondrial dysfunction: a proof-of-concept study

**DOI:** 10.1186/s12967-023-04049-y

**Published:** 2023-03-16

**Authors:** Rongjin Yang, Xiaomeng Zhang, Yunyun Zhang, Yingfan Wang, Man Li, Yuancui Meng, Jianbang Wang, Xue Wen, Jun Yu, Pan Chang

**Affiliations:** 1 Department of Cardiology, The Second Affiliated Hospital of Xi’an Medical University, Xi’an, 710038 Shaanxi China; 2 Department of Cardiology, The 989th Hospital of the People’s Liberation Army Joint Logistic Support Force, 2 Huaxia West Road, Luoyang, 471000 China; 3 Department of Cardiology, Xijing Hospital, Air Force Medical University, 169 Changle West Road, Xi’an, 710032 China; 4 Clinical Experimental Center, The Affiliated Xi’an International Medical Center Hospital, Northwest University, Xi’an, 710100 China

**Keywords:** Diabetic cardiomyopathy, DLST, Grpel2, Mitochondrial dysfunction, Nr2f6

## Abstract

**Background:**

Diabetic cardiomyopathy (DCM) has been considered as a major threat to health in individuals with diabetes. GrpE-like 2 (Grpel2), a nucleotide exchange factor, has been shown to regulate mitochondrial import process to maintain mitochondrial homeostasis. However, the effect and mechanism of Grpel2 in DCM remain unknown.

**Methods:**

The streptozotocin (STZ)-induced DCM mice model and high glucose (HG)-treated cardiomyocytes were established. Overexpression of cardiac-specific Grpel2 was performed by intramyocardial injection of adeno-associated virus serotype 9 (AAV9). Bioinformatics analysis, co-immunoprecipitation (co-IP), transcriptomics profiling and functional experiments were used to explore molecular mechanism of Grpel2 in DCM.

**Results:**

Here, we found that Grpel2 was decreased in DCM induced by STZ. Overexpression of cardiac-specific Grpel2 alleviated cardiac dysfunction and structural remodeling in DCM. In both diabetic hearts and HG-treated cardiomyocytes, Grpel2 overexpression attenuated apoptosis and mitochondrial dysfunction, including decreased mitochondrial ROS production, increased mitochondrial respiratory capacities and increased mitochondrial membrane potential. Mechanistically, Grpel2 interacted with dihydrolipoyl succinyltransferase (DLST), which positively mediated the import process of DLST into mitochondria under HG conditions. Furthermore, the protective effects of Grpel2 overexpression on mitochondrial function and cell survival were blocked by siRNA knockdown of DLST. Moreover, Nr2f6 bond to the Grpel2 promoter region and positively regulated its transcription.

**Conclusion:**

Our study provides for the first time evidence that Grpel2 overexpression exerts a protective effect against mitochondrial dysfunction and apoptosis in DCM by maintaining the import of DLST into mitochondria. These findings suggest that targeting Grpel2 might be a promising therapeutic strategy for the treatment of patients with DCM.

**Supplementary Information:**

The online version contains supplementary material available at 10.1186/s12967-023-04049-y.

## Introduction

Diabetic cardiomyopathy (DCM), which is a serious microvascular complication of diabetes, is a major cause of mortality in individuals with diabetes worldwide [1]. DCM is characterized by cardiac dysfunction and adverse structural remodeling, including decreased contractile function, decreased diastolic function, cardiac hypertrophy, interstitial fibrosis, mitochondrial dysfunction and cardiomyocyte apoptosis [2]. These characteristics of DCM eventually result in heart failure, increasing cardiovascular morbidity and mortality in patients with diabetes [2, 3]. However, the initiation and progression of DCM have not been thoroughly investigated, and effective therapies to prevent the development of DCM remain extremely limited.

Mitochondria are a major source of cellular energy production, and mitochondrial dysfunction is a central event in DCM [4]. Although mitochondria have a dedicated genome that encodes 13 mitochondrial proteins, more than 90% of mitochondrial proteins are encoded by the nuclear genome and require import into mitochondria in mammals [5]. Increasing evidence suggests that the mitochondrial import process plays a vital role in the maintenance of mitochondrial homeostasis in the heart under physiological and pathological conditions [6]. Disruption of the mitochondrial import process leads to impaired proteostasis, decreased ATP production, harmful ROS accumulation and excessive mitochondrial fission, ultimately causing cellular apoptosis [6, 7]. Mitochondrial heat shock protein 70 (mtHSP70) is considered to be a core component of the presequence translocase-associated motor (PAM) [8]. GrpE-like 2 (Grpel2), a nucleotide exchange factor, interacts with mtHSP70 to facilitate the transport of peptide-containing proteins from the inner membrane into the mitochondrial matrix [9]. Previous studies have reported that Grpel2 is involved in mitochondrial stress responses in many tissues [10, 11]. For example, Grpel2 senses oxidative stress and forms dimers that enhance the activity of mtHSP70 to ensure proper mitochondrial protein import and folding [11]. In addition, in glioma cells, Grpel2 ablation stimulates autophagy and senescence, which suppress cell growth [10]. Importantly, our previous study reported that Grpel2 could alleviate myocardial ischaemia/reperfusion injury by inhibiting MCU-mediated mitochondrial calcium ([Ca^2+^]_m_) overload [12]. Given that Grpel2 is a crucial regulator of mitochondrial homeostasis in the heart, it is important to explore the role of Grpel2 in cardiac dysfunction and adverse structural remodeling in DCM.

In the present study, we investigated the role of Grpel2 in the initiation and progression of DCM. We observed that the downregulation of Grpel2 might be a critical factor contributing to heart dysfunction and structural remodeling in DCM. We identified Grpel2 overexpression as a potential therapeutic strategy for DCM. Furthermore, we explored the underlying transcriptional and molecular mechanisms by which Grpel2 regulates normal mitochondrial bioenergetics, oxidative stress and cardiomyocyte survival in the heart during diabetes.

## Materials and methods

### Animals

All animal experiments were carried out according to the National Institutes of Health Guidelines on the Use of Laboratory Animals (NIH Publication, 8th Edition, 2011) and were approved by the Animal Care Committee of the Second Affiliated Hospital of Xi'an Medical University. All male C57BL/6 J mice were maintained in a temperature-controlled environment at 22 ± 2 °C with a 12 h light–dark cycle and free access to food and water. A total number of 108 mice were included in this study.

### Experimental diabetes model

Diabetes mellitus was induced in male 8-week-old C57BL/6 J mice with consecutive daily intraperitoneal injections of streptozotocin (STZ, 50 mg/kg dissolved in 0.1 mol/l citrate buffer, pH 4.5, S0130, Sigma‒Aldrich, USA) for 5 days, as previously described [13]. On the 7th day after the STZ injection, the mice were considered diabetic mice in subsequent experiments if their fasting blood glucose levels were > 11.1 mmol/l. The control mice were intraperitoneally injected with citrate buffer (vehicle). Mice were used for subsequent experiments for 12 weeks post-induction.

### Quantitative real-time PCR (qRT-PCR)

Total RNA was extracted with TRIzol reagent (15596026, Thermo Fisher Scientific, USA) and was reverse-transcribed to cDNA via a PrimeScript™ RT Reagent Kit with gDNA Eraser (RR047A, Takara, Japan). qRT-PCR was performed using SYBR® Premix Ex Taq™ II (RR820A, Takara, Japan) on an Applied Biosystems ABI Prism machine according to the manufacturer’s protocols. Gene expression was detected by using the standard comparative CT method. *β-actin* was used as an internal control to normalize gene expression. The primer sequences were as follows:

**Table:** 

Gene	Forward	Reverse
*Grpel2*	GAGGACCCTCCTGATGGACT	TAATGGTGCGGCCATGAAGT
*β-actin*	AACAGTCCGCCTAGAAGCAC	CGTTGACATCCGTAAAGACC
*Anp*	GATTTCAAGAACCTGCTAGACC	AGTTTGCTTTTCAAGAGGGC
*Bnp*	CACCCAAAAAGAGTCCTTCG	CAACAACTTCAGTGCGTTAC
*Col1a1*	GATGGATTCCCGTTCGAGTA	CGCTGTTCTTGCAGTGATAG
*Col3a1*	ATGGTGGTTTTCAGTTCAGC	GCCTTGAATTCGCCTTCATT
*Tgfb1*	GACCTCAAGAGCTCTAACATCC	GTCATCCACAGACAGAGTAGG
*Nr2f6*	TCCAGGATGGAGGGTCCAAT	CCCACCATCCCACAAGTTCA

### Western blotting analysis

Cells or tissues were homogenized in RIPA lysis buffer supplemented with protease and phosphatase inhibitors (78440, Thermo Fisher  Scientific, USA). After centrifugation at 4 °C for 10 min at 10,000 × g, the protein concentration of the supernatant was determined with a BCA Protein Assay Kit (23225, Thermo Fisher  Scientific, USA). A total of 15–30 μg of protein from each sample was subjected to electrophoresis on 12% SDS‒PAGE gels and then transferred to PVDF membranes. The membranes were blocked in 5% nonfat milk for 1 h and then incubated with primary antibodies overnight at 4 °C. After incubation with HRP-conjugated IgG secondary antibodies for 1 h at room temperature, protein bands were detected using an enhanced chemiluminescence detection kit (Millipore, USA). ImageLab software version 5.1 (Bio-Rad, USA) was used to quantify the protein band intensity. The following primary antibodies were used as followed: β-actin (1:10,000, 4970S, CST, USA); DLST (1:10,000, Ab177934, Abcam, USA); Grpel2 (1:1000, PA5-54723, Invitrogen, USA); Nr2f6 (1:10,000, Ab137496, Abcam, USA); TOMM20 (1:10,000, 42406, CST, USA).

### Intramyocardial adeno-associated virus injection

Adeno-associated virus serotype 9 (AAV9), under the control of a cardiac troponin T promoter (cTnT), was designed and constructed by Tsingke Biotechnology Co., Ltd. (Beijing, China). The AAV9 vectors were suspended in PBS at approximately 3.0*10^11^ PFU/ml. All male mice were anaesthetized with inhaled 2.5% isoflurane and maintained with 2.0% isoflurane during the surgery. After exposing the heart, 10 μl of AAV9 vector was injected into four different sites of the free wall of the left ventricle, as previously described [12]. 12 weeks after AAV injection, transfection efficiency was measured by Western blotting and qRT-PCR analysis. Then, the mice underwent STZ or vehicle injection as described above.

### Echocardiography and hemodynamics

Echocardiography was performed in M-mode with a VEVO 2100 echocardiography system (VisualSonics Inc., Canada) as previously described [14]. During the procedure, mice were anaesthetized using 2.5% isoflurane and maintained with 2% isoflurane. Heart rate was measured by a continuous electrocardiogram monitoring system under conscious or anesthetic condition. Heart images were viewed in the long-axis and short-axis between the two papillary muscles to determine the left ventricular systolic and diastolic motion profile. The left ventricular ejection fraction (LVEF), fraction shortening (LVFS), systolic internal dimension (LVISD) and left ventricular diastolic internal dimension (LVIDD) were automatically calculated with echocardiography software. During M-mode echocardiography analysis in the short axis, LV end-diastolic posterior wall thickness (LVPWd) were obtained at the time of the apparent maximal LV diastolic dimension, and LV end-systolic posterior wall thickness (LVPWs) was obtained at the time of the most anterior systolic excursion of the posterior wall. The end-systolic posterior wall thickening (LVESWT) = (LVPWs − LVPWd)/LVPWs × 100, a marker of regional left ventricle systolic function. The early mitral diastolic wave/late mitral diastolic wave ratio (E/A ratio) were measured by Doppler echocardiography using VEVO 2100 software to evaluate diastolic function as previous described [15].

Haemodynamic parameters were measured by a Millar Mikro-tip-pressure catheter (Millar Instruments, Houston, TX, USA) as previously described [16]. After anaesthetized using 2.5% isoflurane, the right carotid artery was cannulated with a high-fidelity catheter transducer. Haemodynamic parameters were recorded and analysed. The LV end diastolic pressure–volume relationship (EDPVR) and LV end-diastolic pressure (LVEDP) were measured to evaluated cardiac diastolic function.

All measurements were performed by a researcher who was blinded to the experimental groupings.

### Biochemical and histological analysis

Blood glucose levels were measured with an ACCU-CHEK Active Blood Glucose Meter using blood from the tail vein. Plasma insulin levels were determined using a commercial ELISA kit (EZRMI-13K, Millipore, USA) according to the manufacturer’s instructions. After excision, the hearts were fixed with 10% neutral buffered formalin for 24 h, embedded in paraffin, and cut into 5 μm slices. Standard haematoxylin and eosin (H&E) staining (G1120, Solarbio, China) was performed according to the manufacturers’ protocol. Cardiomyocyte number were measured on light microscopy images as previously described [17]. FITC-labelled wheat germ agglutinin staining (WGA staining, W11261, Thermo Fisher, USA) was used to detect the cross-sectional area of the cardiomyocytes per the manufacturer’s protocol. Masson’s trichrome staining (G1346, Solarbio, China) was used for quantification of myocardial interstitial fibrosis. The size of the fibrotic area was calculated as the ratio of the fibrotic area to the left ventricle area. The results were quantified using ImageJ software (NIH, USA).

### Transmission electron microscopy (TEM)

The left ventricular wall was cut into 1–2 mm wide strips perpendicular to its long axis. These strips were fixed with 2.5% glutaraldehyde in 0.1 mol/l phosphate buffer (pH 7.4, 4 °C) for 24 h and postfixed with 1% osmium tetroxide in 0.1 mol/l sodium cacodylate buffer (pH 7.4) for 1 h. After dehydration and embedding in spur resin, the samples were cut into 80 nm-thick sections. All images were obtained with a transmission electron microscope (JEM-1230, JEOL Ltd., Japan) at 80 kV. Mitochondrial images were obtained at magnifications of 28,000 and 98,000 and analysed with ImageJ software in a blinded fashion.

### Cardiomyocyte isolation, culture and transfection

Primary neonatal mouse cardiomyocytes (NCMs) were isolated as previously described [18]. Briefly, the left ventricular tissue was removed from 1 to 3-day-old C57/BL6J mice. The ventricular tissues were minced thoroughly and digested with collagenase type II (1 mg/ml, 17101015, Gibco, Thermo Fisher) for 3 min at 37 °C (4–6 times). After differential plating to remove fibroblasts, the NCMs were cultured in Dulbecco’s modified Eagle’s medium (DMEM) containing 20% foetal bovine serum (FBS) and 1% penicillin–streptomycin for 48 h.

After plating, the NCMs were incubated with high-glucose medium (30 mmol/l glucose, HG) for 48 h to mimic diabetic cardiomyopathy in vivo. The NCMs were treated with normal-glucose medium (5.5 mmol/l glucose, NG) as a control group.

The NCMs were infected with adenovirus Ad-vector (Ad-EV, MOI: 1:50), Ad-Nr2f6 (MOI: 1:50) or Ad-Grpel2 (MOI: 1:50) in complete DMEM for 8 h, and the medium with adenovirus was replaced with fresh complete DMEM. After 48 h of adenoviral transfection, the efficiency of gene overexpression and knockdown was determined by Western blotting and qRT-PCR analysis. Then, the NCMs were subjected to NG or HG treatment for another 48 h.

All recombinant adenovirus and siRNA were constructed by Tsingke Biotechnology Co., Ltd. (Beijing, China). The sequences of siRNAs used in this study are listed below:

**Table:** 

siRNA	Sense	Antisense
siCtrl:	5′-TTCTCCGAACGTGTCACGT-3′	5′-ACGTGACACGTTCGGAGAA-3′
siGrpel2:	5′-GCGGCTCTTTGATGCAAAT-3′	5′-ATTTGCATCAAAGAGCCGC-3′
siDLST	5′-GAUAUUGAACGGACCAUUA-3′	5′-UAAUGGUCCGUUCAAUAUC-3′
siNr2f6	5′-GGUCCAACCGUGACUGUCA-3′	5′-UGACAGUCACGGUUGGACC-3′

### Measurement of the mitochondrial oxygen consumption rate (OCR)

The mitochondrial OCR of NCMs was measured with an XF24 Extracellular Flux Analyser (Agilent Seahorse Bioscience, USA) as previously described [19]. In brief, NCMs were plated into XF24 Seahorse plates at 160,000 cells/well for 36 h and then infected with adenoviruses for 48 h. After the NCMs were exposed to NG or HG conditions, the mitochondrial OCR was determined according to the manufacturer’s protocols. The employed working concentrations of the inhibitors were as follows: oligomycin, 0.6 μM; trifluoromethoxy carbonyl cyanide phenylhydrazone (FCCP), 0.75 μM; antimycin A, 2 μM; and rotenone 1 μM. Basal respiration, maximal respiration, ATP production and spare respiration capacity were calculated by using XF Cell Mito Stress Test Generator software (Agilent Seahorse Bioscience, USA). All OCR measurements were normalized to protein concentrations.

### Measurement of mitochondrial membrane potential

After NG or HG treatment, cardiomyocytes were harvested and incubated with JC-1 (C2003S, Beyotime, China) at 37 °C for 30 min in the dark. The results were analysed within 1 h by flow cytometry. When the mitochondrial membrane potential is high, JC-1 accumulates in the mitochondrial matrix and forms J-aggregates, which can emit red fluorescence. When the mitochondrial membrane potential is low, JC-1 is a monomer that can produce green fluorescence. The analysis of mitochondrial membrane potential is presented as the aggregate (red fluorescence) ratio as previously described [20].

### Detection of ROS content in cardiomyocytes

The total cellular and mitochondrial ROS contents in frozen heart sections were evaluated by DHE staining (10 μM, S0063, Beyotime, China) and staining with the fluorescent probe MitoSOX (100 mM, M36008, Thermo Fisher Scientific, USA), respectively, as previously described [21]. Images were captured with a confocal laser scanning microscope (Nikon, Japan) and analysed with ImageJ software (NIH, USA).

Total cellular ROS in NCMs were detected by a ROS/Superoxide Detection Assay Kit (Ab139476, Abcam, USA) as previously described via a microplate reader [22]. The mitochondrial ROS in NCMs were detected by staining with the fluorescent probe MitoSOX (100 mM, M36008, Thermo Fisher Scientific, USA) following the manufacturers’ protocols via flow cytometry analysis with a BD FACS Aria II flow cytometer.

The Total Glutathione Peroxidase (GPx) Assay Kit with NADPH (S0058, Beyotime, China) and Lipid Peroxidation Malondialdehyde (MDA) Assay Kit (Ab118970, Abcam, USA) were used according to the manufacturer’s instructions to detect cardiomyocyte GPx activity and MDA levels and further assess oxidative stress levels.

### Cell apoptosis assay

The apoptosis rate in primary cardiomyocytes was determined by flow cytometry analysis using an Annexin V-FITC/propidium iodide (PI) apoptosis detection kit (C1062S, Beyotime, China). Apoptosis in heart tissues was measured by terminal deoxynucleotidyl transferase dUTP nick end labelling (TUNEL) staining (C1090, Beyotime, China). All procedures were performed as previously described [23]. Apoptosis levels were calculated by dividing the number of TUNEL-positive nuclei by the total number of 4′,6-diamidino-2-phenylindole (DAPI)-positive nuclei. All images were obtained with a confocal laser scanning microscope (Nikon, Japan) and analysed in a blinded fashion. A caspase 3 activity kit (BC3830, Solarbio, China) was also used to detect myocardial apoptosis.

### ATP detection

Total ATP production was detected with an ATP Assay Kit (S0027, Beyotime, China) following the protocols provided by the manufacturer as previously described [24]. Briefly, NCMs and heart tissues were homogenized and lysed in ice lysis buffer. After centrifugation at 4 °C at 12,000 × g for 20 min, 10 μl of the supernatant or standard ATP solution was incubated with the ATP probe in 96-well plates at room temperature in the dark. The results were detected with a luminometer (BioTek Epoch, USA).

### Measurement of cell viability

Cell viability was evaluated with a CCK-8 assay kit (C0005, Topscience, China) as previously described [25]. Briefly, after NG or HG treatment, NCMs were incubated with fresh complete medium containing 10% CCK-8 for 2 h. The absorbance at 450 nm was measured to indicate cell viability.

### Isolation of mitochondria

The complete process of mitochondria isolation was performed at 4 °C using a Cell Mitochondria Isolation Kit (C3601, Beyotime, China) as previously described [26]. NCMs were washed twice with ice-cold PBS and homogenized in ice-cold isolation buffer for 10 min. The homogenates were centrifuged at 1000 × g for 10 min. The supernatant was centrifuged at 11,000 × g for 10 min to pellet mitochondria. Mitochondria-enriched fractions were washed with PBS at 3000 × g for 10 min and resuspended in mitochondrial storage buffer for storage at − 80 °C.

### Co-immunoprecipitation (Co-IP)

NCMs were homogenized with ice-cold IP lysis buffer (25 mM Tris–HCl pH 7.4, 150 mM NaCl, 1% NP-40, 1 mM EDTA and 5% glycerol, 87787, Thermo Fisher Scientific, USA) supplemented with a protease and phosphatase inhibitor cocktail (5872, CST, USA). Subsequent procedures were performed as previously described [27]. The homogenates were centrifuged at 10,000 × g for 10 min. Then, 1 mg of the supernatant was incubated with 1 μg of antibodies for sufficient immunoprecipitation at 4 °C overnight and incubated with protein A/G magnetic beads for another 3 h at 4 °C. After the beads were discarded, the supernatant was used for Western blotting analysis. Forty micrograms of cell lysate were used for the positive control and loading control.

### Chromatin immunoprecipitation (ChIP) assay

ChIP assays were performed using a ChIP Plus Enzymatic Chromatin IP Kit (9003, CST, USA) following the manufacturer’s protocols [21]. Briefly, NCMs were fixed with formaldehyde, and the chromatin was sheared into fragments. Then, the fragmented chromatin was incubated and precipitated with an Nr2f6 antibody (#NBP1-04676, Novus, USA) and protein G magnetic beads at 4 °C. DNA released from the precipitates was analysed by qRT-PCR. IgG was used as the negative control.

### Statistical analysis

All values are presented as the mean ± standard error (SD). All data were obtained in three or more independent experiments. The normality of the distribution for all data was examined using the Shapiro–Wilk normality test. If the data passed the normality assumption, statistical significance was determined by an unpaired, 2-tailed Student’s *t* test (two groups) or one-way ANOVA, followed by Tukey’s post hoc test (> 2 groups); otherwise, the data were analysed with the Mann–Whitney *U* test (two groups) or Kruskal–Wallis test with Dunn’s post hoc test (> 2 groups). Survival analysis was performed by Log-rank Mantel-Cox testing. Correlation analysis was performed using the Pearson correlation test. All statistical analyses were carried out by using GraphPad Prism 8.0 software (GraphPad Software, La Jolla, USA). A value of *p* < 0.05 was considered statistically significant.

## Results

### Cardiac-specific overexpression of Grpel2 effectively alleviates diabetes-induced cardiac dysfunction

**Fig. 1:**
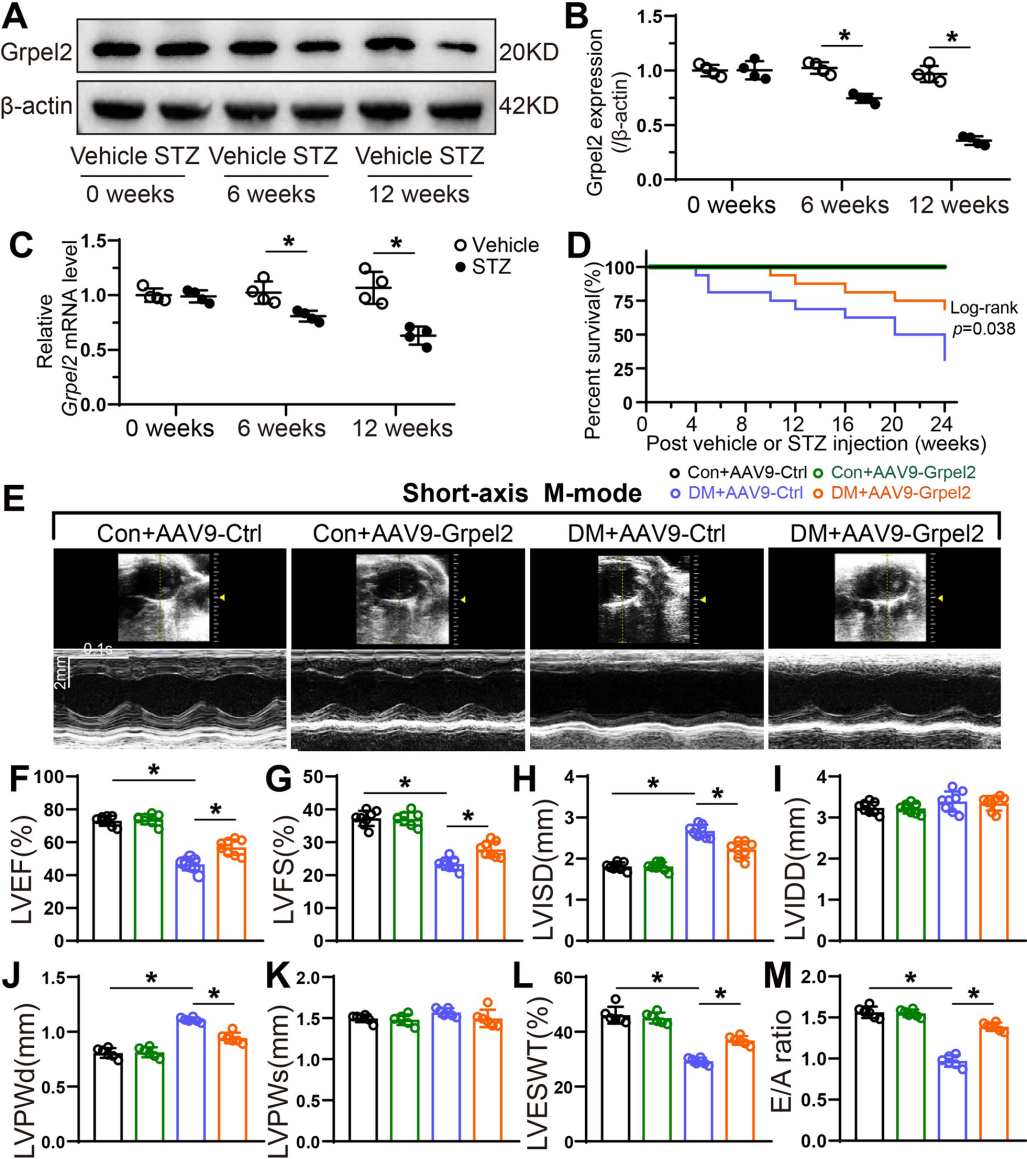
Grpel2 was downregulated in the diabetic heart, and cardiac overexpression of Grpel2 attenuated the contractile dysfunction induced by STZ injection. **A**, **B** Western blotting and quantitative analysis of Grpel2 protein expression in the heart tissues of mice at 0, 6 and 12 weeks after vehicle or STZ injection (*n* = 4/group). **C** Quantitative real-time PCR of Grpel2 mRNA levels in heart tissues from mice at 0, 6 and 12 weeks after vehicle or STZ injection (*n* = 4/group). **D** Survival curve of mice intramyocardially injected with AAV9-Ctrl or AAV9-Grpel2 after vehicle or STZ injection (*n* = 16/group). **E** Representative short-axis M-mode echocardiographic images from mice intramyocardially injected with AAV9-Ctrl or AAV9-Grpel2 12 weeks after vehicle or STZ injection. **F**–**L** Quantification of left ventricular ejection fraction (LVEF, **F**), fraction shortening (LVFS, **G**), systolic internal dimension (LVISD, **H**), diastolic internal dimension (LVIDD, **I**), end-diastolic posterior wall thickness (LVPWd, **J**), end-systolic posterior wall thickness (LVPWs, **K**), and end-systolic posterior wall thickening (LVESWT, **L**) by short-axis M-mode echocardiography (*n* = 6–8/group). **M** Quantitative analysis of the early mitral diastolic wave/late mitral diastolic wave ratio (E/A ratio) by Doppler echocardiography (*n* = 6/group). Data are presented as the mean ± SD. Data in **B** and **C** were analysed with an unpaired, 2-tailed Student’s *t* test. Data in **D** were analysed with Log-rank Mantel-Cox testing. Other data were analysed by one-way ANOVA, followed by Tukey’s post hoc test. *p < 0.05

To gain insight into the role of Grpel2 in the diabetic cardiomyopathy (DCM), we first assessed Grpel2 expression by Western blotting and qRT-PCR analysis in a STZ-induced diabetic mouse model. We found that Grpel2 protein expression and mRNA levels were both decreased in the hearts of mice 6 weeks after STZ injection compared to vehicle injected group. The hearts showed an even greater downregulation of Grpel2 protein and  mRNA levels at 12 weeks after STZ injection (Fig. 1a–c). To characterize the effect of diabetes on cardiomyocyte Grpel2 levels in vitro, we also isolated primary neonatal mouse cardiomyocytes (NCMs) and subjected them to high-glucose (30 mmol/l glucose, HG) conditions to mimic diabetes in vivo. We found that Grpel2 protein expression and mRNA levels were significantly decreased 48 h after HG treatment (Additional file 1: Fig. S1A–C). An adeno-associated virus serotype 9 (AAV9) was designed and intramyocardially injected to overexpress cardiac Grpel2 expression to investigate the relationship between Grpel2 and DCM. A set of experimental analyses was carried out as shown in Additional file 1: Fig. S2A. As a result, intramyocardial injection of AAV9-Grpel2 successfully increased Grpel2 protein expression and mRNA levels in the hearts of the control or diabetic mice (Additional file 1: Fig. S2B–D). Compared with the control mice, the diabetic mice had decreased serum insulin, increased blood glucose levels and lower survival rate (Fig. 1d and Additional file 1: Fig. S3A, B). Although Cardiac-specific overexpression of Grpel2 had no significant effects on serum insulin or blood glucose levels, the diabetic mice injected with AAV9-Grpel2 showed a higher survival rate compared with mice injected with AAV9-Ctrl (Fig. 1d and Additional file 1: Fig. S3A, B). To evaluate the effect of Grpel2 on cardiac systolic function in diabetic mice, the left ventricular ejection fraction (LVEF), left ventricular fractional shorting (LVFS), left ventricular systolic internal diameter (LVISD) and left ventricular diastolic internal diameter (LVIDD) were measured using echocardiography from short-axis and long-axis views 12 weeks after vehicle or STZ injection. Our data revealed that diabetes-induced cardiac systolic dysfunction, including decreased LVEF and LVFS as well as increased LVISD, was ameliorated in AAV9-Grpel2-injected diabetic mice (Fig. 1e–i and Additional file 1: Fig. S4A–C). Furthermore, LV end-diastolic posterior wall thickness (LVPWd) was significantly increased in diabetic mice, which was attenuated by AAV9-Grpel2 injection, whereas no significant differences were observed in LV end-systolic posterior wall thickness (LVPWs) among the groups (Fig. 1j, k). And the LV end-systolic posterior wall thickening (LVESWT) was markedly decreased in diabetic mice, which was improved by cardiac-specific Grpel2 overexpression (Fig. 1l). Moreover, haemodynamic parameters and Doppler echocardiography were performed to evaluated the LV diastolic function. LV end diastolic pressure–volume relationship (EDPVR) and LV end-diastolic pressure (LVEDP) were significantly increased in diabetic mice compared with control mice. Grpel2 overexpression markedly decreased EDPVR and LVEDP in diabetic mice (Table 1). Moreover, the diabetic mice injected with AAV9-Grpel2 showed improved diastolic function as evidenced by increased early mitral diastolic wave/late mitral diastolic wave ratio (E/A ratio) (Fig. 1m). And Grpel2 overexpression had no evident effects on heart rate under conscious or anesthetic condition (Additional file 1: Fig. S5). In summary, our data indicates that Grpel2 expression is downregulated in the diabetic heart and that cardiac-specific overexpression of Grpel2 alleviates diabetes-induced cardiac contractile and diastolic dysfunction.

**Table 1:** Pressure–volume loop data

	Con + AAV9-Ctrl n = 6	Con + AAV9-Grpel2 n = 6	DCM + AAV9-Ctrl n = 6	DCM + AAV9-Grpel2 n = 6
ESPVR, mmHg/ml	23.46 ± 2.39	22.19 ± 2.45	15.54 ± 1.39*	18.79 ± 2.04^#^
EDPVR, mmHg/ml	0.041 ± 0.005	0.054 ± 0.006	0.156 ± 0.004*	0.106 ± 0.003^#^
Tau, ms	7.76 ± 0.29	8.19 ± 0. 54	10.78 ± 1.09*	9.49 ± 1.01^#^
+ dP/dt, mmHg/s	5930 ± 131	5971 ± 143	2569 ± 214*	3742 ± 325^#^
− dP/dt, mmHg/s	3562 ± 176	3687 ± 217	2196 ± 235*	2957 ± 167^#^
LVESP, mmHg	128.56 ± 11.71	130.33 ± 9.81	85.33 ± 8.59*	104.17 ± 6.24^#^
LVEDP, mmHg	4.67 ± 0.83	5.35 ± 1.07	12.12 ± 1.06*	7.48 ± 1.15^#^

Data are mean ± SD

ESPVR, end systolic pressure–volume relationship; EDPVR, end diastolic pressure–volume relationship; LV, left ventricle; ESP, end-systolic pressure; EDP, end-diastolic pressure

*p < 0.05 versus Con + AAV9-Ctrl, ^#^*p* < 0.05 versus DCM + AAV9-Ctrl, using one-way ANOVA, followed by Tukey’s post-hoc test

### Cardiac-specific Grpel2 overexpression alleviated adverse cardiac remodeling in the diabetic heart

**Fig. 2:**
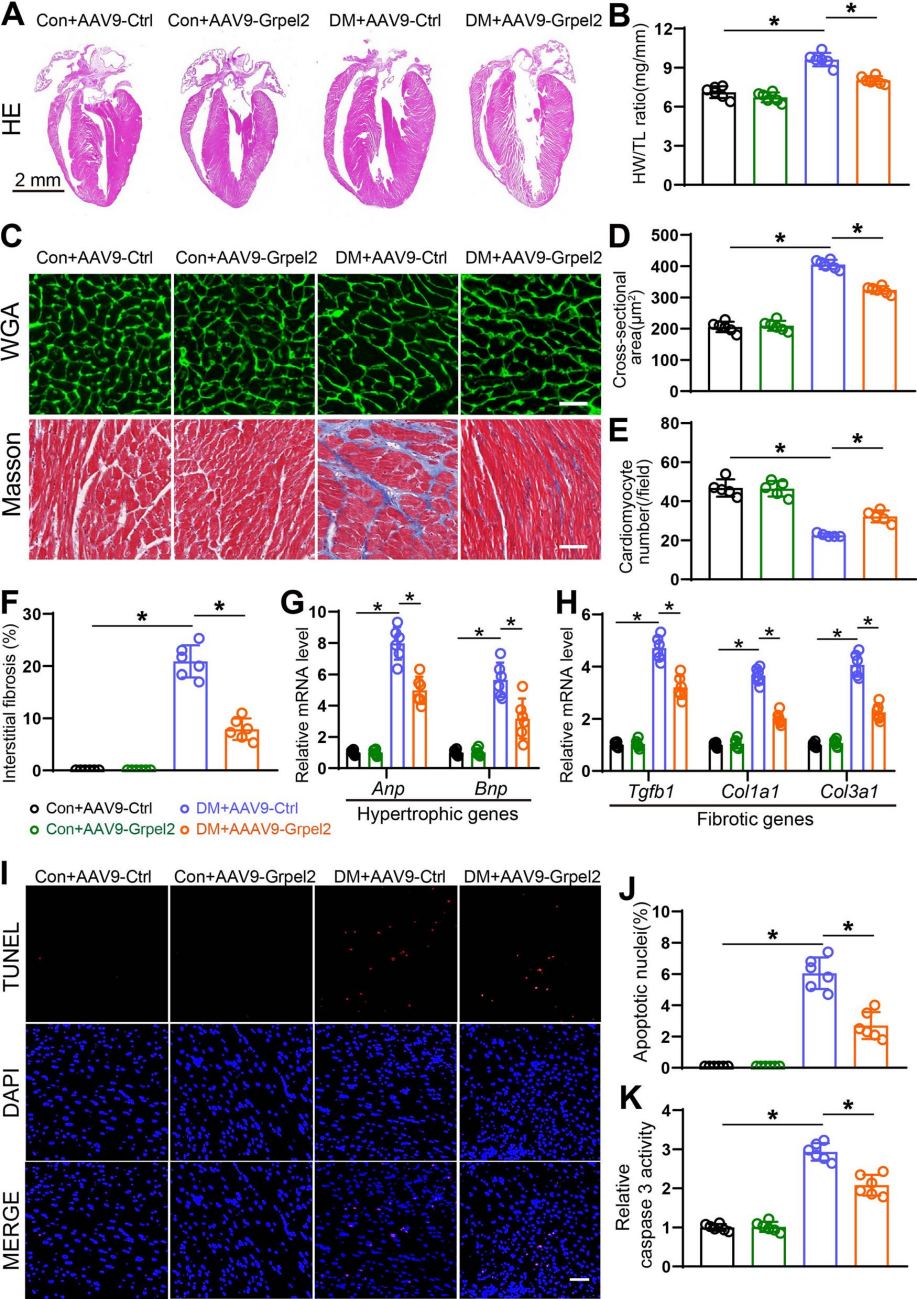
Overexpression of cardiac Grpel2 alleviated heart remodeling in DCM. **A**, **B** Representative images of haematoxylin and eosin (HE) staining of cardiac sections (*n* = 6/group). Scale bar = 2 mm. **B** Quantification of the heart weight/tibia length (HW/TL) ratio (*n* = 6/group). **C** Representative images of wheat germ agglutinin (WGA) staining (top) and Masson trichrome staining (bottom) of cardiac sections. Scale bars: 25 μm (top) and 50 μm (bottom). **D**, **E** Quantitative analysis of the cross-sectional area of cardiomyocytes (**D**) and number of cardiomyocytes (**E**) by WGA staining. **F** Quantitative analysis of interstitial fibrosis by Masson trichrome staining (*n* = 6/group). **G**, **H** Quantitative real-time PCR (qRT-PCR) of the mRNA levels of hypertrophy-associated genes (**G**) and fibrosis-associated genes (**H**) in the left ventricle of mice (*n* = 6/group). **I**, **J** Representative images and quantitative analysis of TUNEL staining of cardiac sections (*n* = 6/group). Scale bar: 50 μm. **K** Quantification of relative caspase 3 activity in the left ventricle of mice (*n* = 6/group). Data are presented as the mean ± SD. Data were analysed by one-way ANOVA, followed by Tukey’s post hoc test. *p < 0.05

Since pathological myocardial hypertrophy and excess cardiac fibrosis are the most prominent features of DCM [28, 29], we next explored the potential role of Grpel2 in pathological cardiac remodeling induced by diabetes. There were no significant differences in heart pathology among the control mice. The hearts of diabetic mice exhibited significant cardiac remodeling, which was exemplified by enlarged hearts, elevated ratios of heart weight to tibia length, reduced cardiomyocyte number, increased cardiomyocyte cross-sectional area and increased interstitial fibrosis (Fig. 2a–f). Importantly, these pathological changes were efficiently ameliorated in the hearts of diabetic mice receiving AAV9-Grpel2 injection (Fig. 2a–f). Moreover, we detected the mRNA levels of common hypertrophic markers, such as *Anp* and *Bnp,* and fibrotic markers, such as *Tgfb1*, *Col1a1* and *Col3a1*. There were no differences in the mRNA levels of common hypertrophic markers or fibrotic markers among control mice. However, the mRNA levels of these markers were significantly decreased in Grpel2-overexpressing mice compared to mice intramyocardially injected with AAV9-Ctrl after STZ treatment (Fig. 2g, h). In addition, we detected cardiomyocyte apoptosis, a common feature of the diabetic heart. As we expected, cardiomyocyte apoptosis, as determined by TUNEL staining and caspase 3 activity assays, was significantly decreased in diabetic mice injected with AAV9-Grpel2 compared with diabetic mice injected with AAV9-Ctrl (Fig. 2i–k). Collectively, our data indicated that cardiac-specific Grpel2 overexpression significantly ameliorates cardiac structure remodeling and cardiomyocyte apoptosis in diabetic mice.

### Grpel2 overexpression attenuated mitochondrial morphological disorder and oxidative stress in the diabetic heart

**Fig. 3:**
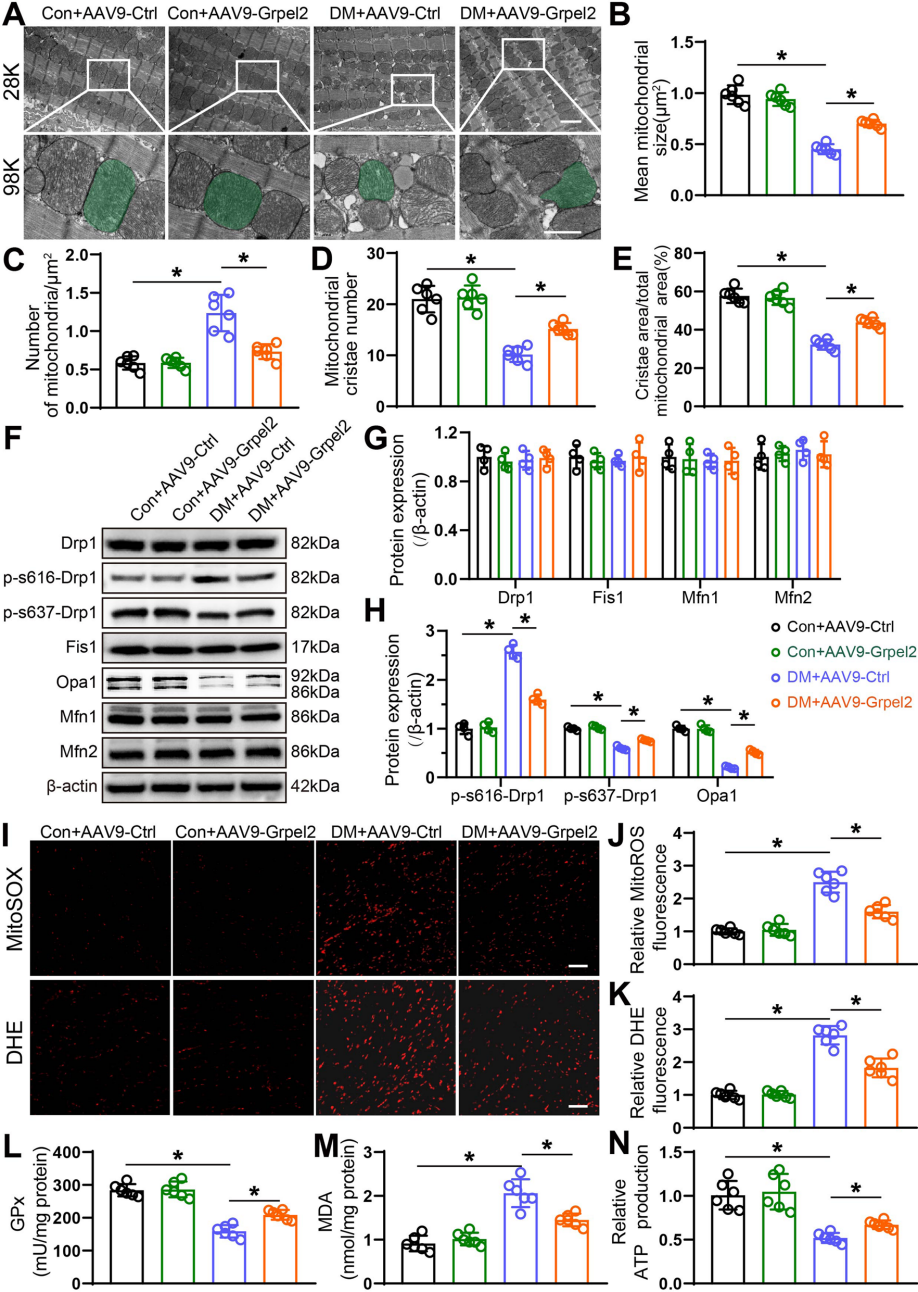
Grpel2 overexpression protected against cardiomyocyte mitochondrial dysfunction in the diabetic heart. **A** Representative transmission electron microscopy images of the left ventricle of mice (magnification: 28,000 × [28 K] and 98,000 × [98 K]). Scale bars: 1 μm (top) and 0.5 μm (bottom). **B**–**E** Quantitative analysis of the mean mitochondrial size (**B**), number of mitochondria per μm^2^ (**C**), number of mitochondrial cristae per mitochondrial area (**D**) and cristae area per mitochondrial area (**E**) (*n* = 6/group). **F**–**H** Western blotting and quantitative analysis of mitochondrial fission related proteins (Drp1and Fis1) and fusion-related proteins (Opa1, Mfn1 and Mfn2) in the left ventricle of mice (*n* = 4/group). **I** Representative images of MitoSOX staining (top) and DHE staining (bottom) of cardiac sections. Scale bars: 50 μm (up and down). **J**, **K** Quantitative analysis of relative mitoROS fluorescence (**J**) by MitoSOX staining and relative DHE fluorescence (**K**) by DHE staining (*n* = 6/group). **L**, **M** Quantitative analysis of glutathione peroxidase (GPx) content (**L**) and malondialdehyde (MDA) content (**M**) in the left ventricle of mice (*n* = 6/group). **N** Quantitative analysis of the relative ATP content in heart lysates (*n* = 6/group). Data are presented as the mean ± SD. Data were analysed by one-way ANOVA, followed by Tukey’s post hoc test. *p < 0.05

Excessive mitochondrial fission and oxidative stress lead to cardiomyocyte apoptosis, contributing to diabetic cardiomyopathy [15, 30]. As shown in the transmission electron microscopy (TEM) images, cardiomyocytes of the diabetic heart presented excessive mitochondrial fission, as indicated by a significantly larger mean mitochondrial size, an increased number of mitochondria per μm^2^ and a decreased number and area of mitochondrial cristae per mitochondrial area. No significant differences in mitochondrial morphology were observed in control mice (Fig. 3a–f). Grpel2 overexpression alleviated diabetes-induced mitochondrial morphological abnormalities, as reflected by the larger mean mitochondrial size, decreased number of mitochondria per μm^2^, increased number of mitochondrial cristae per mitochondrial area and increased cristae area per mitochondrial area (Fig. 3a–f). Moreover, of the mitochondrial fission proteins, Phospho-Ser-616-Drp1 expression was markedly increased and Phospho-Ser-637-Drp1 was significantly decreased, while Drp1 and Fis1 remained unchanged in diabetic hearts compared with control hearts. Of the mitochondrial fusion proteins, Mfn1 and Mfn2 remained unchanged, but the Opa1 expression was significantly decreased in the diabetic hearts compared with control hearts. Importantly, cardiac-specific Grpel2 overexpression markedly increased Opa1 and Phospho-Ser-637-Drp1 expressions, and significantly decreased Phospho-Ser-616-Drp1 expression in the diabetic hearts (Fig. 3f–h). We next investigated the effect of Grpel2 on oxidative stress in the diabetic heart. Mitochondrial ROS levels and total ROS levels were detected by MitoSOX staining and DHE staining, respectively. As anticipated, total cellular and mitochondrial ROS levels were markedly increased in the diabetic heart compared to the control heart (Fig. 3i–k). Moreover, Grpel2 overexpression significantly decreased the total and mitochondrial ROS contents compared to those of diabetic mice intramyocardially injected with AAV9-Ctrl (Fig. 3i–k). Furthermore, we also determined the GPx and MDA contents to evaluate cardiac oxidative stress. Similarly, there were no evident differences in control mice, while Grpel2 overexpression significantly increased the GPx content and decreased the MDA content in diabetic mice (Fig. 3l, m). Excessive mitochondrial fission and oxidative stress cause defects in cardiomyocyte mitochondrial ATP bioenergetics in the diabetic heart [31]. We found that Grpel2 overexpression significantly increased ATP levels in the diabetic heart (Fig. 3n). Therefore, these data indicate that Grpel2 overexpression attenuates diabetes-induced excessive mitochondrial fission and oxidative stress in the diabetic heart.

### Grpel2 overexpression alleviated HG-induced apoptosis and mitochondrial dysfunction in NCMs

**Fig. 4:**
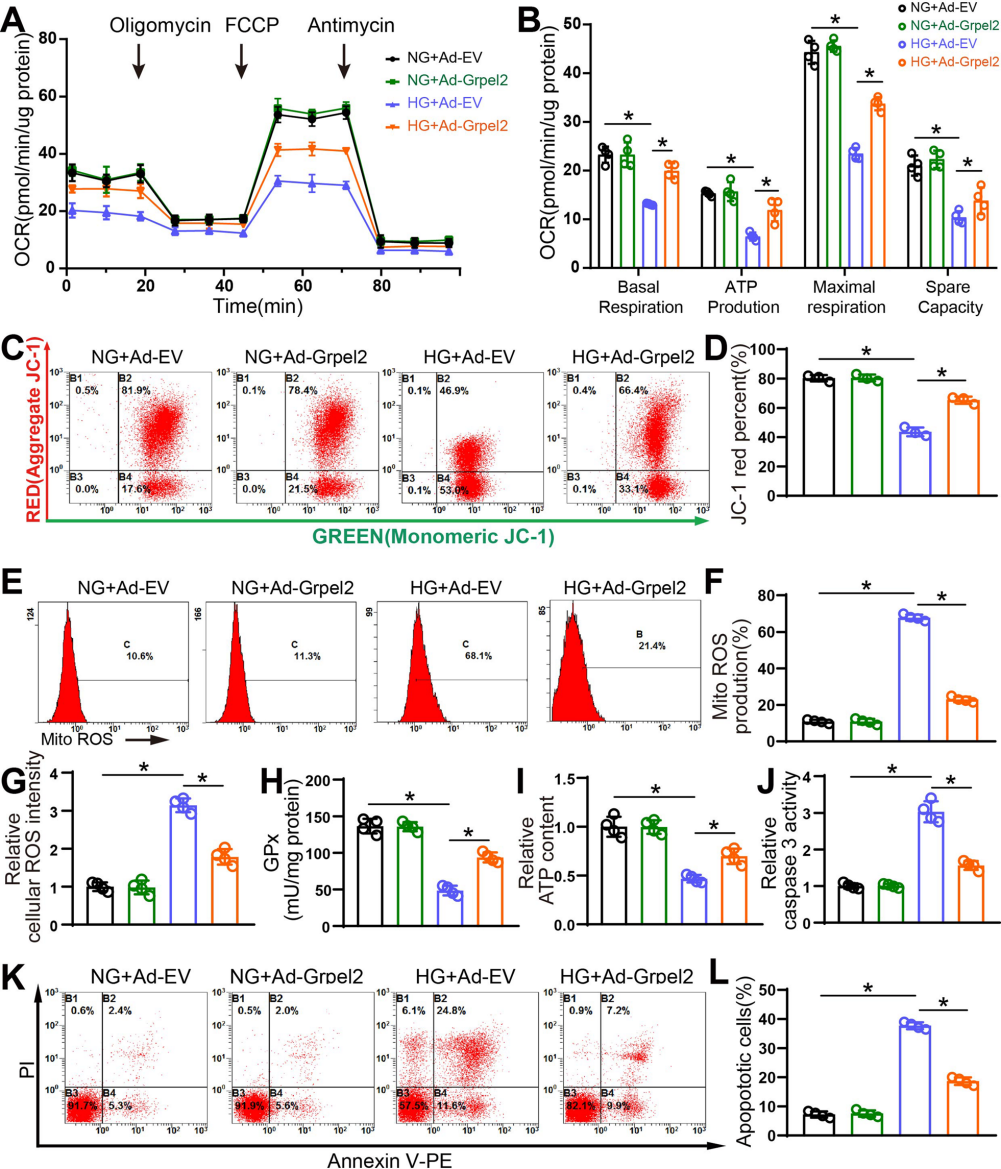
Grpel2 overexpression attenuated HG-induced mitochondrial dysfunction in vitro. **A**, **B** Oxygen consumption rate (OCR) and associated quantitative analysis of mitochondrial respiratory function in NCMs (*n* = 4/group). **C**, **D** Flow cytometry analysis and quantification of mitochondrial membrane potential by JC-1 staining in NCMs (*n* = 4/group). **E**, **F** Flow cytometry analysis and quantification of mitochondrial ROS content by MitoSOX staining in NCMs (*n* = 4/group). **G** Quantification of total intracellular ROS intensity in NCMs (*n* = 4/group). **H** Quantitative analysis of glutathione peroxidase (GPx) content in NCMs (*n* = 4/group). **I** Quantitative analysis of the relative ATP content in NCMs (*n* = 4/group). **J** Quantification of relative caspase 3 activity in control or Grpel2-overexpressing NCMs under NG or HG conditions (n = 4/group). **K**, **L** Flow cytometry analysis and quantification of apoptotic cells by Annexin V-FITC and propidium iodide (PI) staining (*n* = 4/group). Data are presented as the mean ± SD. Data were analysed by one-way ANOVA, followed by Tukey’s post hoc test. *p < 0.05

We also designed a recombinant adenovirus encoding Grpel2 (Ad-Grpel2) to overexpress Grpel2 in NCMs. Transfection with Ad-Grpel2 resulted in successful overexpression of Grpel2 protein and mRNA in NCMs (Additional file 1: Fig. S6A–C). We further detected the effects of Grpel2 on mitochondrial function in vitro. We first assessed the effect of Grpel2 on mitochondrial respiratory capacity by measuring the oxygen consumption rate (OCR, a classical indicator of mitochondrial function). Compared to the NG-treated NCMs, NCMs treated with HG exhibited decreased mitochondrial respiratory capacities, including basal respiration, maximal respiration, ATP production and spare respiration capacity (Fig. 4a, b). There was no change in NCMs infected with Ad-EV or Ad-Grpel2 under NG conditions. Notably, Grpel2 overexpression significantly enhanced the mitochondrial respiratory capacities of HG-treated NCMs (Fig. 4a, b). Decreased mitochondrial membrane potential is also a key feature of mitochondrial dysfunction. As expected, Grpel2 overexpression markedly increased the mitochondrial membrane potential in HG-treated NCMs (Fig. 4c, d). We next determined the contents of total and mitochondrial cellular ROS to further evaluate mitochondrial oxidative stress. Similar to the results above, Grpel2 overexpression did not change the ROS content under NG conditions but markedly decreased both mitochondrial ROS and total cellular ROS in HG-treated NCMs (Fig. 4e–g). Furthermore, the GPx content was increased in NCMs infected with Ad-Grpel2 under HG conditions (Fig. 4h). Importantly, Grpel2 overexpression markedly increased ATP content under HG conditions (Fig. 4i). And Grpel2 overexpression significantly decreased cardiomyocyte apoptosis, as detected by Annexin V/PI staining and caspase 3 activity assays, under HG conditions (Fig. 4j–l). Collectively, these results suggest that Grpel2 is involved in maintaining cardiomyocyte apoptosis and mitochondrial homeostasis under HG conditions.

### Grpel2 positively mediated the process of DLST import into mitochondria

**Fig. 5:**
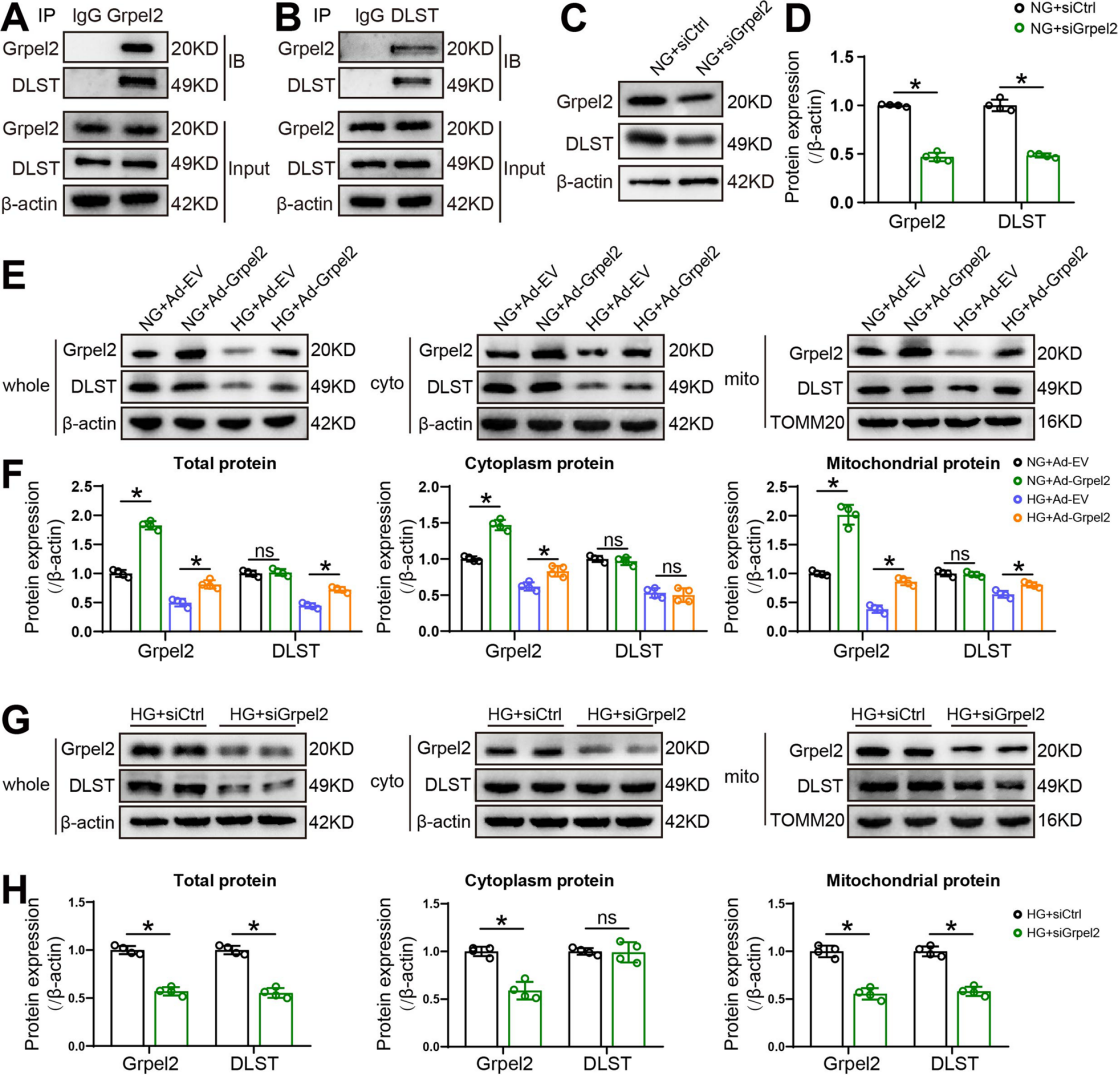
Grpel2 promotes the process of DLST import into mitochondria. **A** Co-immunoprecipitation (IP) analysis of DLST with Grpel2 in whole-cell lysates of NCMs. **B** Co-IP analysis of Grpel2 with DLST in whole cell lysates of NCMs. **C**, **D** Western blotting and quantitative analysis of Grpel2 and DLST protein expression in NCMs infected with siCtrl or siGrpel2 under NG conditions (*n* = 4/group). **E**, **F** Western blotting and quantitative analysis of Grpel2 and DLST protein expression in the whole cell, cytoplasm or mitochondrial lysates of NCMs infected with Ad-EV or Ad-Grpel2 under NG or HG conditions (*n* = 4/group). **G**, **H** Western blotting and quantitative analysis of Grpel2 and DLST protein expression in the whole cell, cytoplasm or mitochondrial lysates of NCMs infected with siCtrl or siGrpel2 under HG conditions. Data are presented as the mean ± SD. Data in **H** were analysed by an unpaired, 2-tailed Student’s *t* test. Other data were analysed by one-way ANOVA, followed by Tukey’s post hoc test. *p < 0.05; ns, not significant

Given the important roles of Grpel2 in maintaining cardiac function and structure in DCM, we next investigated the underlying molecular mechanism responsible for the regulation of Grpel2-mediated mitochondrial function and cardiomyocyte survival. Previous studies revealed that Grpel2 facilitated mitochondrial protein import, and most of the possible interaction partners of Grpel2 were metabolic enzymes, including dehydrogenases of the tricarboxylic acid (TCA) cycle [11]. Among those potential interactors, dihydrolipoyl succinyltransferase (DLST) is one of unique interactors with high confidence [11]. We first found that there were interactions between Grpel2 and DLST, as detected by co-immunoprecipitation (IP) (Fig. 5a, b). Furthermore, knockdown of Grpel2 by siRNA decreased the protein expression of DLST under NG conditions (Fig. 5c, d). Considering that DLST is a nucleus-encoded protein and requires import into mitochondria, we suggested that Grpel2 mediates the import process of DLST into mitochondria. We found that Grpel2 overexpression had no effect on DLST expression in mitochondrial or cytoplasmic lysates under NG condition. However, Grpel2 overexpression increased DLST expression in mitochondrial lysates, but did not affect DLST expression in cytoplasmic lysates under HG conditions (Fig. 5e, f). And knockdown of Grpel2 had no effect on DLST expression in cytoplasmic lysates but decreased DLST expression in mitochondrial lysates under HG conditions (Fig. 5g, h). In summary, these data revealed that Grpel2 interacted with DLST and may be involved in the process of DLST import into mitochondria under HG conditions.

### Grpel2 overexpression protected mitochondrial function by maintaining mitochondrial import of DLST

**Fig. 6:**
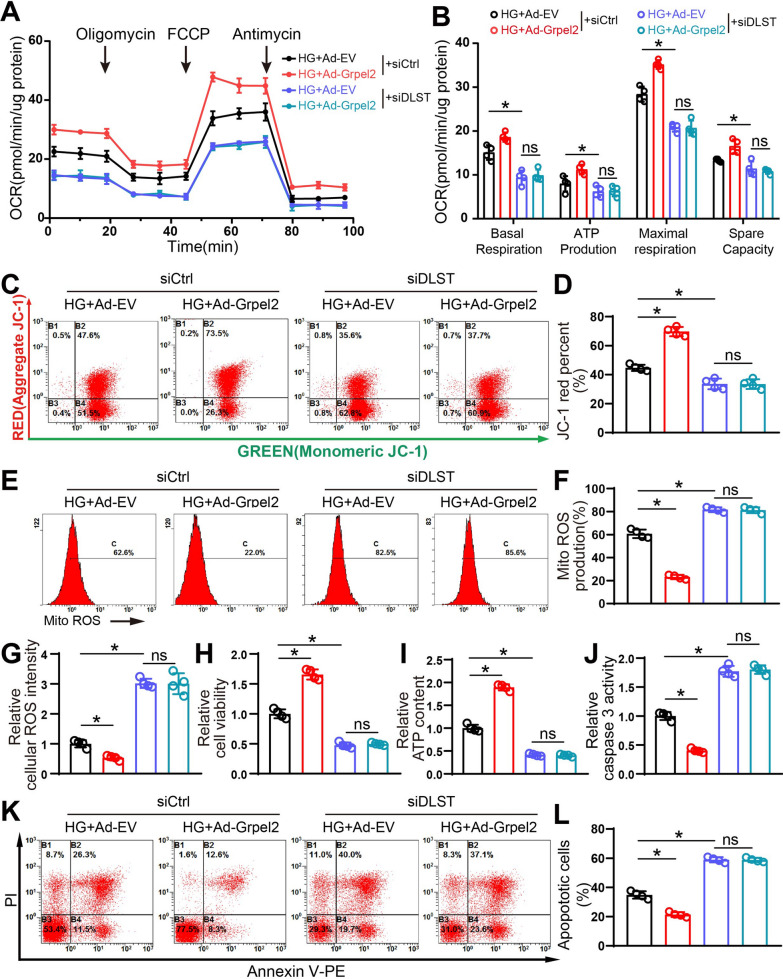
Grpel2 overexpression alleviates HG-induced mitochondrial dysfunction and apoptosis via mitochondrial DLST expression. **A**, **B** OCR and associated quantitative analysis of mitochondrial respiratory function in NCMs (*n* = 4/group). **C**, **D** Flow cytometry analysis and quantification of mitochondrial membrane potential by JC-1 staining in NCMs (*n* = 4/group). **E**, **F** Flow cytometry analysis and quantification of mitochondrial ROS content by MitoSOX staining in NCMs (*n* = 4/group). **G** Quantification of total intracellular ROS intensity in NCMs (*n* = 4/group). **H** Quantitative analysis of relative cell viability in NCMs (*n* = 4/group). **I** Quantitative analysis of the relative ATP content in NCMs (*n* = 4/group). **J** Quantification of relative caspase 3 activity in control or Grpel2-overexpressing NCMs under NG or HG conditions (n = 4/group). **K**, **L** Flow cytometry analysis and quantification of apoptotic cells by Annexin V-FITC and propidium iodide (PI) staining (*n* = 4/group). Data are presented as the mean ± SD. Data were analysed by one-way ANOVA, followed by Tukey’s post hoc test. *p < 0.05; ns, not significant

To verify whether DLST is essential for the mitochondria-protective effects of Grpel2 in DCM, NCMs infected with Ad-EV or Ad-Grpel2 were also subjected to siRNA to block DLST expression under HG conditions. siRNA significantly decreased DLST expression (Additional file 1: Fig. S7A–C). As expected, Grpel2 overexpression significantly attenuated HG-induced mitochondrial dysfunction, as indicated by elevated mitochondrial respiratory capacities, increased mitochondrial membrane potential, decreased mitochondrial ROS production and decreased cellular ROS production (Fig. 6a–g). Interestingly, the protective effects of Grpel2 overexpression on mitochondrial function were almost blocked by DLST knockdown in NCMs under HG conditions. Even when Grpel2 was overexpressed, NCMs with DLST knockdown still exhibited more severe impaired mitochondrial function under HG conditions, including decreased mitochondrial respiratory capacities, decreased mitochondrial membrane potential and increased mitochondrial ROS production (Fig. 6a–g). Similarly, downregulation of DLST also markedly decreased cell viability and ATP contents, and increased cardiomyocyte apoptosis under HG conditions (Fig. 6i–l). All the protective effects of Grpel2 overexpression on cell survival were eliminated by DLST knockdown in NCMs under HG conditions (Fig. 6i–l). Overall, the protective effects of Grpel2 overexpression on mitochondrial function and cell survival were dependent on the expression of DLST.

### Nr2f6 regulates the transcription of Grpel2 by directly binding the promoter of Grpel2

**Fig. 7:**
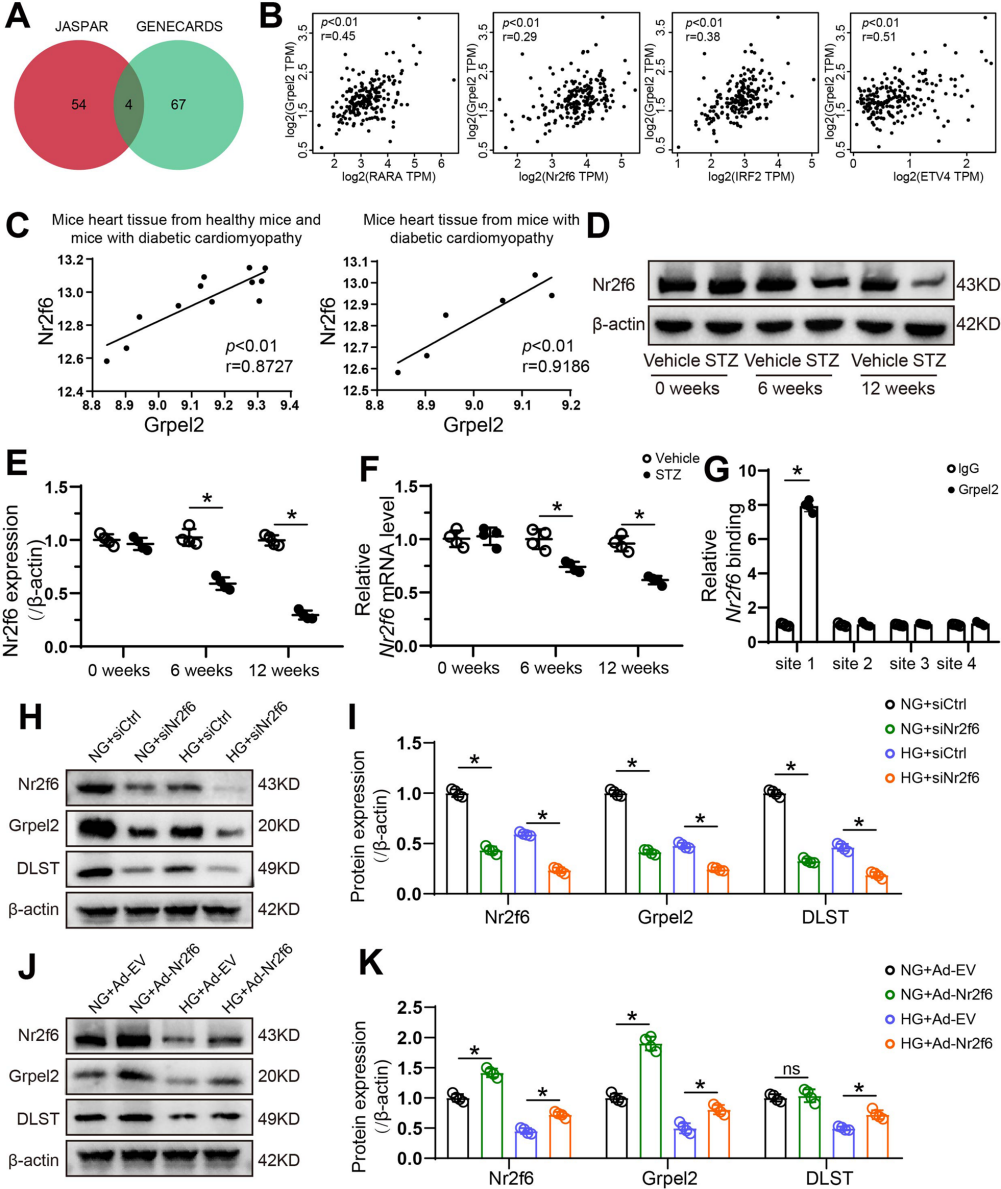
Nr2f6 directly binds to the promoter of Grpel2 and regulates Grpel2 expression. **A** Venn diagram showing the candidate transcription factors identified in the JASPAR and GENECARDS databases. **B** Scatter plots showing the Pearson correlations for the mRNA levels of Grpel2 and candidate transcription factors in human left ventricular samples from the GTEx database through the GEPIA website. **C** Scatter plots showing Pearson correlation analysis of mRNA levels of Nr2f6 and Grpel2 in control or diabetic heart tissue based on the GEO database (GSE123975). **D**, **E** Western blotting and quantitative analysis of heart Nr2f6 protein expression in the heart tissues of mice at 0, 6 and 12 weeks after STZ injury (*n* = 4/group). **F** qRT-PCR of Grpel2 mRNA levels in heart tissues of mice at 0, 6 and 12 weeks after STZ injury (*n* = 4/group). **G** Chromatin immunoprecipitation (ChIP) and qRT-PCR analysis of the binding of Nr2f6 to the Grpel2 promoter in NCMs (*n* = 4/group). **H**, **I** Western blotting and quantitative analysis of Nr2f6, mitochondrial Grpel2 and mitochondrial DLST protein expression in NCMs infected with siCtrl or siNr2f6 under NG or HG conditions (*n* = 4/group). **J**, **K** Western blotting and quantitative analysis of Nr2f6, and mitochondrial Grpel2 protein expression in NCMs infected with Ad-EV or Ad-Nr2f6 under NG or HG conditions (*n* = 4/group). Data are presented as the mean ± SD. Data in **B**, **C** were analysed by Pearson correlation analysis. Data in **I**, **K** were analysed by one-way ANOVA, followed by Tukey’s post hoc test. Other data were analysed by an unpaired, 2-tailed Student’s *t* test. *p < 0.05; ns, not significant

We next investigated the underlying transcriptional mechanism responsible for regulating Grpel2-mediated mitochondrial function and cardiomyocyte survival. We used the bioinformatics databases JASPAR and GENECARDS to predict candidate transcription factors of human Grpel2 (Fig. 6a). Four potential transcription factors (Nr2f6, RARA, IRF2 and ETV4) appeared in both databases and had significant correlations with Grpel2 in human left ventricular (LV) samples from the GEPIA database (Fig. 7a, b). Furthermore, we analysed a public microarray dataset from the Gene Expression Omnibus (GEO) database to evaluate the correlations between the mRNA expression of Grpel2 and those potential transcription factors in LV samples from normal control mice and diabetic mice. Pearson correlation analysis revealed that only Nr2f6 had a significant positive correlation with Grpel2 (Fig. 7c and Additional file 1: Fig. S8A–C). Importantly, this correlation became more significant in LV samples from only diabetic mice (Fig. 7c). Moreover, both the protein and mRNA levels of Nr2f6 were significantly decreased in diabetic hearts in vivo and in HG-treated NCMs in vitro (Fig. 7d–f and Additional file 1: Fig. S9A–C). We found a direct binding site of Nr2f6 in the Grpel2 promoter region of NCMs by ChIP-qRT-PCR assays (Fig. 6g and Additional file 1: Fig. S9D). In addition, Nr2f6 knockdown via siRNA markedly decreased the mitochondrial Grpel2 protein expression, whereas Nr2f6 overexpression via Ad-Nr2f6 significantly increased the mitochondrial Grpel2 protein expression under NG or HG conditions (Fig. 7h–k). And the mitochondrial DLST expression was consistent with the results in NCMs infected with siGrpel2 or Ad-Grpel2 under NG or HG conditions (Fig. 7h–k). Collectively, our results reveal that Nr2f6 directly binds to the Grpel2 promoter region and positively regulates its transcription.

## Discussion

In the present study, we reported for the first time that Grpel2 is downregulated in DCM induced by STZ. Furthermore, Grpel2 overexpression significantly alleviated heart dysfunction and cardiac remodeling in DCM, including cardiac contractile dysfunction, cardiac diastolic function cardiac hypertrophy, interstitial fibrosis and cardiomyocyte apoptosis. Specifically, Grpel2 overexpression markedly attenuated mitochondrial dysfunction. These results in vivo were also verified in NCMs treated with HG in vitro. Moreover, we discovered that upregulated Grpel2 expression exerted protective effects against HG-induced mitochondrial dysfunction by binding to DLST and modulating its mitochondrial import process. Finally, our study demonstrated that the downregulation of Grpel2 was partially due to a decrease in Nr2f6 expression in HG-treated NCMs. Taken together, these findings suggest that Grpel2 plays a positive role in DCM and that targeting Grpel2 may represent a new therapeutic method for DCM.

The prevalence of diabetes mellitus has been rapidly increasing, and diabetes mellitus is a major threat to human health worldwide [32]. DCM has a high incidence and high mortality in diabetic patients [31]. However, the underlying pathological mechanism of DCM remains unclear. An increasing number of studies have reported that the mitochondrial protein import process is involved in mitochondrial bioenergetics, mitochondrial dynamics and protein quality control. Recent studies revealed that defects in the mitochondrial import process of nucleus-encoded protein subunits result in inhibition of mitochondrial respiratory capacities, decreased mitochondrial membrane potential and reduction of ATP synthesis in many cardiovascular diseases, such as heart failure, ischaemic cardiomyopathy, DCM and hypertension [6]. It is reported that mtHSP70, a central subunit of the presequence translocase-associated motor complex, was decreased in DCM, which coincides with decreased protein import in the diabetic interfibrillar mitochondria subpopulation [33]. However, the mechanisms involved in import process dysfunction have been not entirely clear.

Grpel2, a member of the mtHSP70 chaperone family, is involved in sensing oxidative stress to import precursor proteins in the mitochondrial matrix, thereby maintaining protein quality control and mitochondrial homeostasis [11, 34]. Grpel2 can form disulfide bonds and mediate metabolic adaptation to redox stress in a high-fat-feeding-induced oxidative environment [11]. A previous study reported that Grpel2 ablation significantly increased ROS production and promoted apoptosis by inhibiting the NF-κB pathway in hepatocellular carcinoma [34]. Our previous work indicated that Grpel2 alleviates myocardial ischaemia/reperfusion injury by inhibiting MCU-mediated mitochondrial calcium overload [12]. In this study, we explored the expression of Grpel2 in the diabetic heart and its role in DCM. For the first time, we observed that Grpel2 expression was decreased in the diabetic heart and in HG-treated NCMs. Then, to achieve prolonged overexpression of cardiac-specific Grpel2 in vivo, we designed adeno-associated virus serotype 9 under the control of a specific cTnT promoter. Cardiac Grpel2 overexpression markedly alleviated heart contractile and diastolic dysfunction induced by STZ injection. Moreover, Grpel2 overexpression attenuated cardiac hypertrophy, interstitial fibrosis and apoptosis in STZ-induced DCM. Considering that AAV-targeted gene therapies have low immunogenicity and have been explored in many preclinical and clinical studies, AAV9-Grpel2 treatment to alleviate heart dysfunction might be further validated in diabetic patients, which could be beneficial and provide hope for patients with diabetes.

Mitochondrial dysfunction and apoptosis orchestrate cardiomyocyte survival and death in the pathogenesis of DCM [15]. In the heart under pathological conditions, damaged mitochondria produce excessive harmful ROS, which eventually leads to apoptosis [35]. Mitochondrial homeostasis plays a central role in cardiomyocyte survival in DCM. Mitochondria depend on their necessary protein import machinery to respond to cellular stress. To date, mouse models for studying Grpel2-mediated mitochondrial function in vivo have not been reported, and little is known regarding the effects of Grpel2 on mitochondrial function in DCM. In the present study, we revealed that Grpel2 overexpression by AAV9-Grpel2 injection alleviated diabetes-induced mitochondrial morphological abnormalities and decreased oxidative stress levels in the diabetic heart in vivo. AAV transduction required a relatively high MOI in the cardiomyocytes, generally up to 1000 [36–38]. The MOI of adenoviruses used for the transfection of cardiomyocytes is much lower, generally 50–100 [39]. Moreover, adenoviruses have been widely used to transfect primary neonatal cardiomyocytes in our previous studies [31, 40] Thus, we continue to use adenoviruses in vitro in this study. Transfecting with AAV in primary cardiomyocytes is an alternative way for us in future studies. Our results indicated that Grpel2 overexpression by Ad-Grpel2 alleviated HG-induced mitochondrial dysfunction and apoptosis in NCMs. Overall, we demonstrated a protective role of Grpel2 in regulating mitochondrial oxidative stress in DCM.

To explore the detailed molecular mechanism by which Grpel2 regulates mitochondrial function, NCMs were isolated and infected with adenovirus in vitro*,* Compared with cell lines, primary neonatal mouse cardiomyocytes (NCMs) retain more biological characteristics of the original tissues in vivo. Due to ethical reasons and highly similar functional properties, isolated NCMs have been the most widely used models to study cardiac biology in vitro*.* We focused on the unique interactors of Grpel2 in an available public database [11]. We found that Grpel2 could bind to DLST by co-IP assays. As reported, DLST assists in converting α­ketoglutarate (αKG) into succinyl-CoA in the TCA cycle, which acts as an important entry point for glutamine anaplerosis [41]. Loss of DLST inhibits the electron transport chain via the reduction of NADH levels in human neuroblastoma cells [42]. DLST depletion induces ROS production in triple-negative breast cancer cells [43]. Overexpression of DLST in cardiomyocytes protects the heart against cardiac dysfunction upon pressure overload in vivo [44]. Our data revealed that Grpel2 knockdown decreased mitochondrial DLST expression, and upregulation of Grpel2 could maintain mitochondrial DLST expression under HG conditions. Importantly, the protective effects of Grpel2 overexpression on mitochondrial homeostasis were blocked by DLST knockdown. These results indicated that Grpel2 interacted with DLST and may be involved in facilitating DLST import into mitochondria to maintain mitochondrial function under HG conditions.

We further explored the transcriptional mechanism of Grpel2 gene expression in NCMs. By performing bioinformatics analysis and ChIP assays, we found that Nr2f6 directly bound to the Grpel2 promoter region and regulated the transcription and expression of Grpel2. Nr2f6 belongs to the nuclear receptor (NR) superfamily, whose members directly bind to DNA loci as transcription factors [45]. Many NRs are critical for the development of the nervous system and heart, such as Nr2f1 and Nr2f2 [46]. Nr2f6 plays a crucial role in the regulation of hepatic lipid metabolism by directly binding to the CD36 promoter [47]. Previous studies have suggested that Nr2f6 may positively regulate tumour cell survival and induce cancer progression [48, 49]. An increasing number of studies have reported that Nr2f6 acts as a checkpoint that limits inflammatory tissue damage [50]. Our results revealed that Nr2f6 was downregulated in DCM. If Nr2f6 was knocked down by siRNA or overexpressed by adenovirus, Grpel2 was downregulated or upregulated accordingly. Our results provide the first evidence for understanding the regulatory mechanisms upstream of Grpel2 in the diabetic heart. Based on its effect on Grpel2 expression, Nr2f6 may also serve as a potential therapeutic target for DCM.

There are some limitations of our study. Firstly, since women with DCM also need effective therapies, female mice were not included in the in vivo studies. Secondly, the specific and dynamic process by which Grpel2 facilitates the import of DLST into mitochondria in DCM is still unclear. Thirdly, even though we identified Nr2f6 as a Grpel2 promoter, we cannot exclude other possible transcriptional mechanisms that contribute to Grpel2 expression. Finally, although many conclusions have been drawn on the basis of mouse models in vivo and in vitro, further preclinical and clinical studies are needed to confirm these results. Despite these limitations, we believe that our findings provide important novel insights for understanding the protective roles of Grpel2 and the underlying regulatory mechanisms in DCM.

## Conclusion

**Fig. 8:**
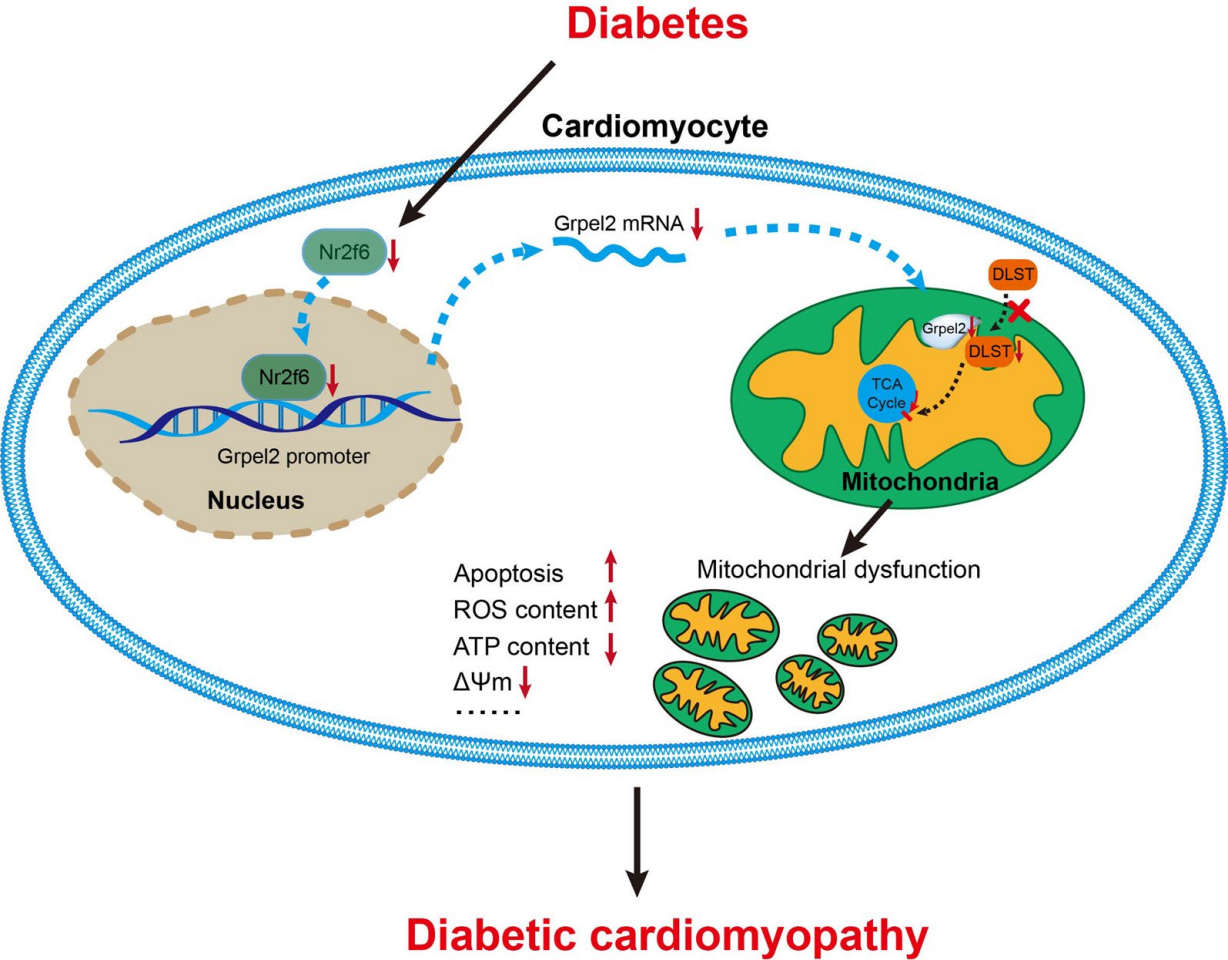
Schematic illustration of a novel molecular mechanism. Schematic illustration of a novel molecular mechanism by which the reduction of Nr2f6-regulated Grpel2 expression results in mitochondrial dysfunction in diabetic heart. Nr2f6 binds to the Grpel2 promoter to promote the Grpel2 expression. Diabetes-induced Nr2f6 reduction decreased the Grpe2 expression, which inhibited the import process of DLST into mitochondria. Afterward, the deficiency of DLST in mitochondria results in mitochondrial dysfunction, including increased ROS content, decreased ATP contents and decreased mitochondrial membrane potential, eventually aggravating DCM

In summary, we uncovered for the first time that Grpel2 markedly attenuated heart dysfunction and cardiac remodeling in DCM by suppressing mitochondrial dysfunction, oxidative stress and aopotosis through Nr2f6 mediation the import of DLST into mitochondria in cardiomyocytes (Fig. 8). These findings suggest that targeting Grpel2 might be a promising therapeutic application for the treatment of patients with DCM.

## Supplementary Information

**Additional file 1.** Supplementary materials
