# Supplementary material for: Grpel2 maintains cardiomyocyte survival in diabetic cardiomyopathy through DLST-mediated mitochondrial dysfunction: a proof-of-concept study
Source: J Transl Med. 2023 Mar 16;21:200. doi: 10.1186/s12967-023-04049-y (PMC10021968; doi:10.1186/s12967-023-04049-y)
Supplement: Supplementary file 1 — Additional file 1. Supplementary materials [file 12967_2023_4049_MOESM1_ESM.docx]

**Supplementary materials**

**Supplementary figure legends**

**
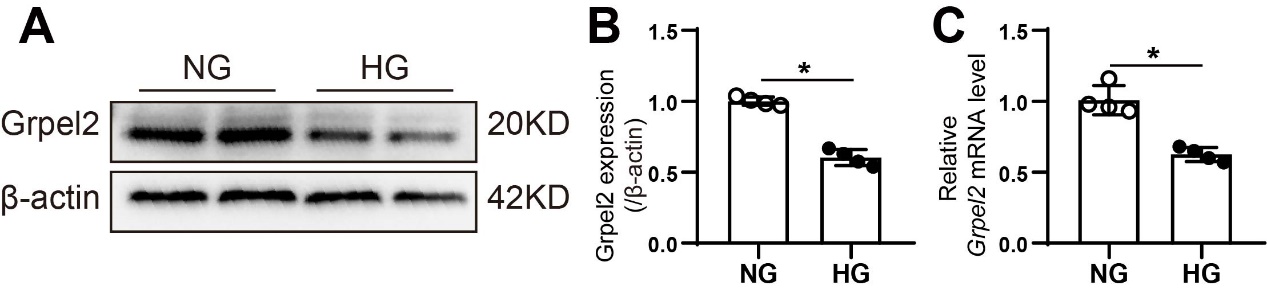
**

**Supplemental Figure S1. Grpel2 expression was downregulated in NCMs under HG conditions.** (*A* and *B*) Western blotting and quantitative analysis of Grpel2 protein expression in primary neonatal mouse cardiomyocytes (NCMs) treated with normal-glucose medium (5.5 mmol/l glucose, NG) or high-glucose medium (30 mmol/l glucose, HG) for 48 hours (*n*=4/group). (C) qRT-PCR of Grpel2 mRNA levels in NCMs treated with NG or HG condition for 48 hours (*n*=4/group). Data were presented as mean ± SD. Data were analyzed by unpaired, 2-tailed Student’s t-test. **P*<0.05.

**
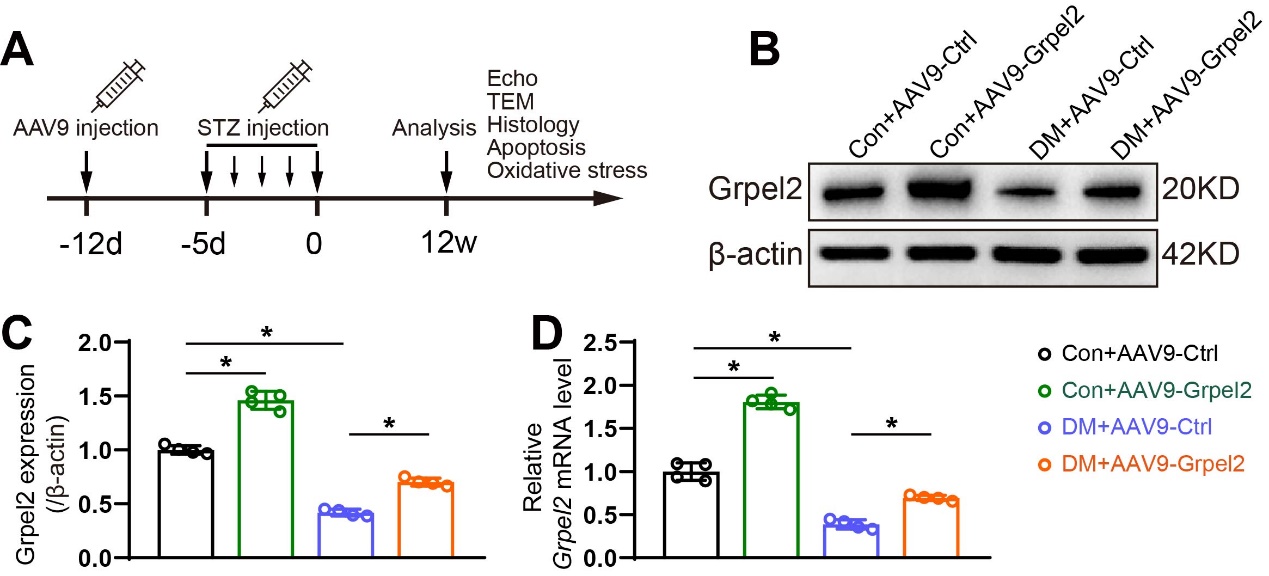
**

**Supplemental Figure S2. Efficiency of Grpel2 overexpression in the hearts.** (*A*) Flowchart of the animal experiments involving adeno-associated virus serotype 9 (AAV9)-Ctrl or AAV9-Grpel2 injection in diabetic mice. (*B and C*) Western blotting and quantitative analysis of Grpel2 protein expression in hearts injected with AA9-Ctrl or AAV9-Grpel2 for 12 weeks (*n*=4/group). (*D*) qRT-PCR of Grpel2 mRNA levels in heart tissues injected with AA9-Ctrl or AAV9-Grpel2 for 12 weeks (*n*=4/group). Data were presented as mean ± SD. Data were analyzed by one-way ANOVA, followed by Tukey’s post hoc test. **P*<0.05.


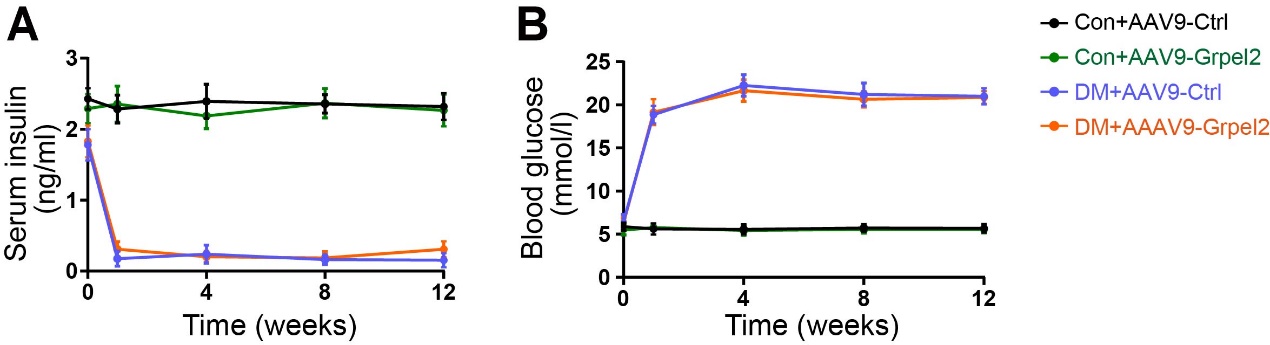


**Supplemental Figure S3. Cardiac Grpel2 overexpression had no effect on serum insulin levels and blood glucose levels.** Mice intramyocardially injected with AA9-Ctrl or AAV9-Grpel2 were subjected to vehicle or STZ treatment for 12 weeks. (*A*) Serum insulin levels in mice at 0, 1, 4, 8 and 12 weeks after vehicle or STZ treatment (*n*=6/group). (*B*) Blood glucose levels in mice at 0, 1, 4, 8 and 12 weeks after vehicle or STZ treatment (*n*=6/group). Data were presented as mean ± SD.


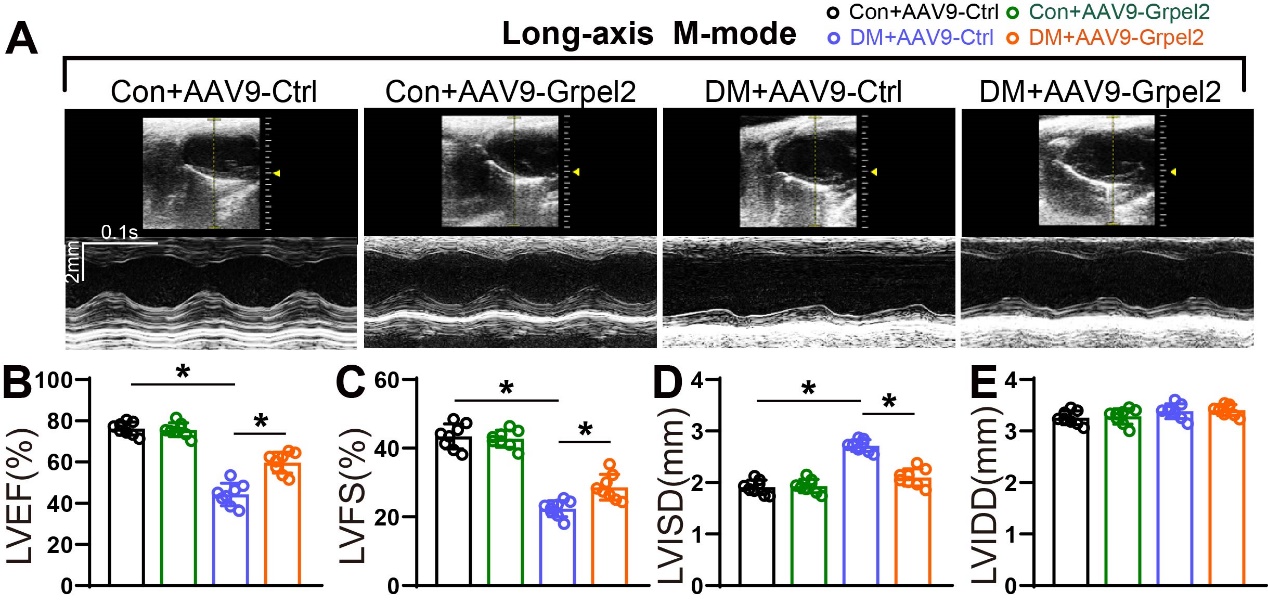


**Supplemental Figure S4. Cardiac Grpel2 overexpression attenuated systolic dysfunction in diabetic mice.** (*A*) Representative long-axis M-mode echocardiographic images from mice intramyocardially injected with AAV9-Ctrl or AAV9-Grpel2 12 weeks after vehicle or STZ injection. (*B-E*) Quantification of left ventricular ejection fraction (LVEF; *B*), left ventricular shortening fraction (LVFS; *C*), left ventricular internal systolic diameter (LVISD; *D*) and left ventricular internal diastolic diameter (LVIDD; *E*) by long-axis M-mode echocardiography (*n*=8/group). Data are presented as the mean ± SD. Data were analysed by one-way ANOVA, followed by Tukey’s post hoc test. **P*<0.05.


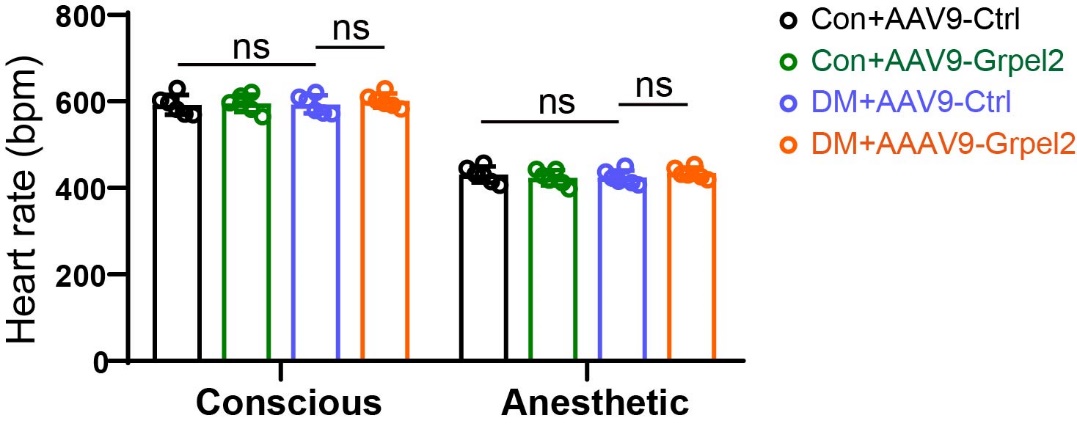


**Supplemental Figure S5. Cardiac Grpel2 overexpression had no effect on heart rate under conscious or anesthetic condition.** Heart rate of mice intramyocardially injected with AAV9-Ctrl or AAV9-Grpel2 under conscious or anesthetic condition 12 weeks after vehicle or STZ injection (*n*=6/group). Data are presented as the mean ± SD. Data were analysed by one-way ANOVA, followed by Tukey’s post hoc test. **P*<0.05.

**
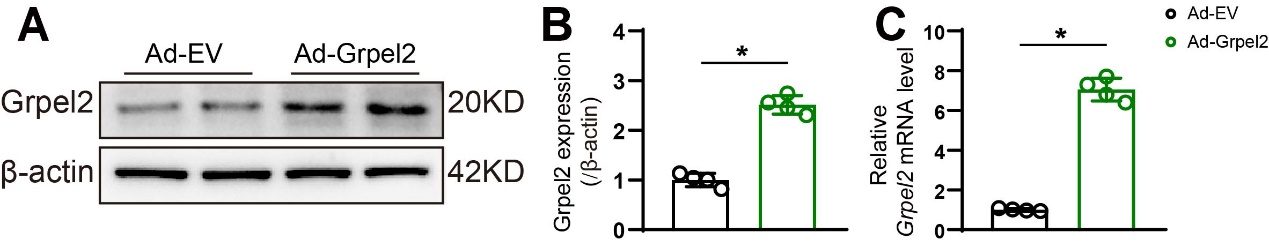
**

**Supplemental Figure S6. Efficiency of Grpel2 overexpression in NCMs.** (*A and B*) Western blotting and quantitative analysis of Grpel2 protein expression in NCMs infected with control adenovirus (Ad-EV) or recombinant adenovirus encoding Grpel2 (Ad-Grpel2) for 48 hours (*n*=4/group). (*C*) qRT-PCR of Grpel2 mRNA levels in NCMs infected with Ad-EV or Ad-Grpel2 for 48 hours (*n*=4/group). Data are presented as mean ± SD. Data were analyzed by unpaired, 2-tailed Student’s t-test. **P*<0.05.

**
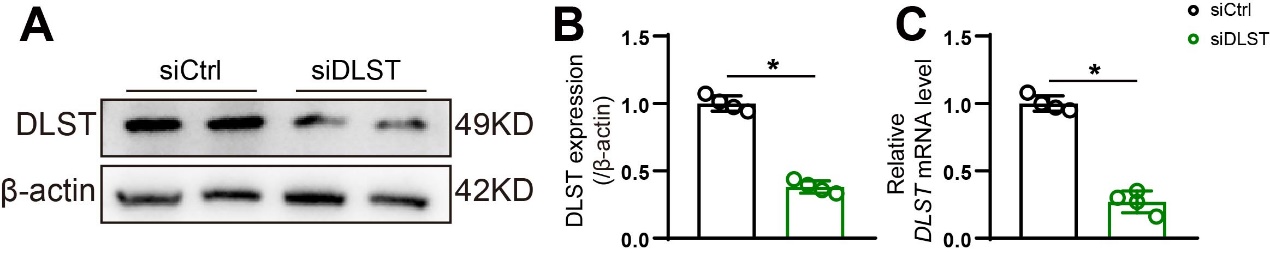
**

**Supplemental Figure S7. Knockdown efficiency of DLST expression in NCMs.** (*A and B*) Western blotting and quantitative analysis of DLST protein expression in NCMs infected with siCtrl or siDLST for 48 hours (*n*=4/group). (*C*) qRT-PCR of DLST mRNA levels in NCMs infected with Ad-EV or Ad-Grpel2 for 48 hours (*n*=4/group). Data are presented as mean ± SD. Data were analyzed by unpaired, 2-tailed Student’s t-test. **P*<0.05.


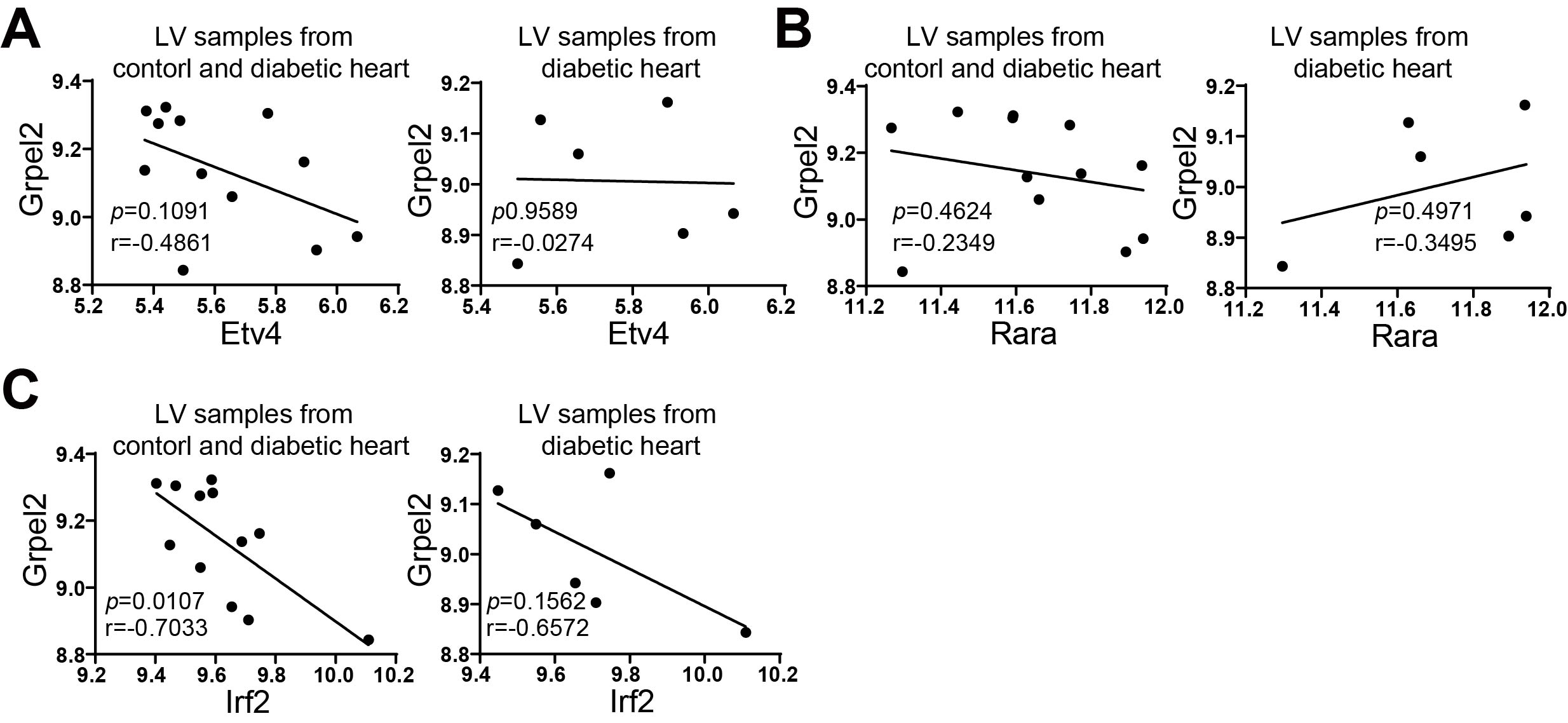


**Supplemental Figure S8. Pearson correlation analysis between the mRNA levels of Grpel2 and candidate transcription factors (Evt4, Rara and Irf2) in LV samples from mice with or without diabetic cardiomyopathy.** Data were analyzed by Pearson correlation analysis.

**
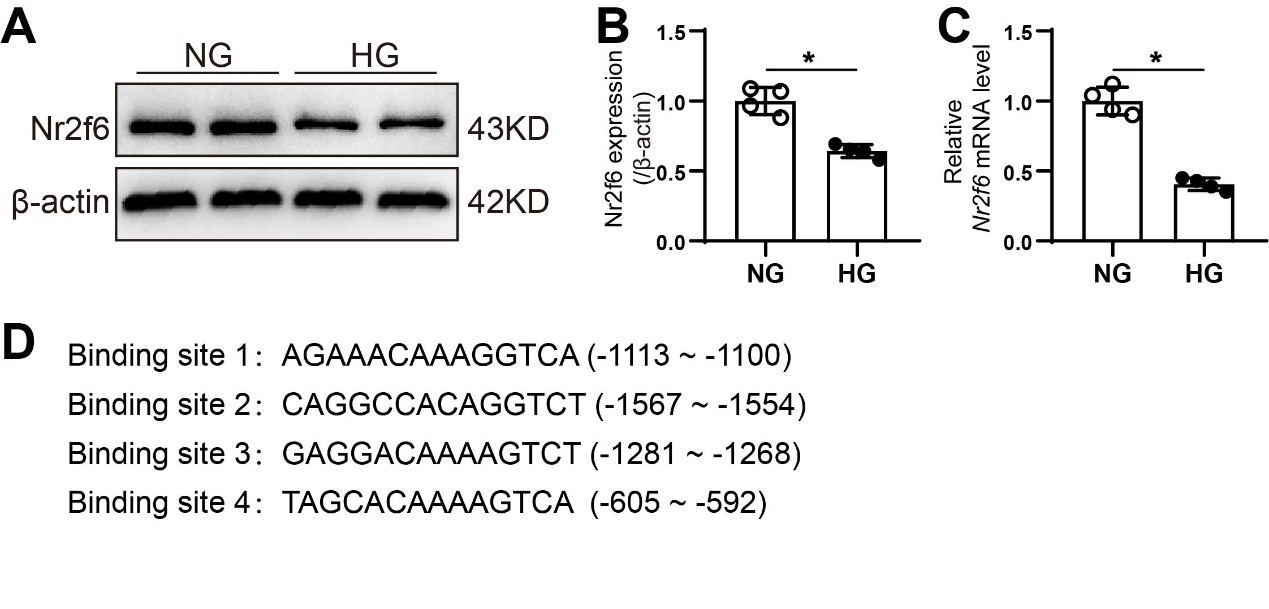
**

**Supplemental Figure S9. Nr2f6 was decreased in HG-induced injury.** (*A and B*) Western blotting and quantitative analysis of Nr2f6 protein expression in NCMs treated with NG or HG conditions for 48 hours (*n*=4/group). (*C*) qRT-PCR of Nr2f6 mRNA levels in NCMs treated with NG or HG conditions for 48 hours (*n*=4/group). (*D*) G) Promoter region of Grpel2 contains potential conserved binding sites for Nr2f6. Data were presented as mean ± SD. Data were analyzed by unpaired, 2-tailed Student’s t-test. **P*<0.05.
